# Metastasis-associated fibroblasts in peritoneal surface malignancies

**DOI:** 10.1038/s41416-024-02717-4

**Published:** 2024-05-23

**Authors:** Cristiano Ramos, Vasileios Gerakopoulos, Rudolf Oehler

**Affiliations:** Department of General Surgery, Division of Visceral Surgery, Medical University of Vienna, Vienna, Austria

**Keywords:** Cancer microenvironment, Tumour heterogeneity

## Abstract

Over decades, peritoneal surface malignancies (PSMs) have been associated with limited treatment options and poor prognosis. However, advancements in perioperative systemic chemotherapy, cytoreductive surgery (CRS), and hyperthermic intraperitoneal chemotherapy (HIPEC) have significantly improved clinical outcomes. PSMs predominantly result from the spread of intra-abdominal neoplasia, which then form secondary peritoneal metastases. Colorectal, ovarian, and gastric cancers are the most common contributors. Despite diverse primary origins, the uniqueness of the peritoneum microenvironment shapes the common features of PSMs. Peritoneal metastization involves complex interactions between tumour cells and the peritoneal microenvironment. Fibroblasts play a crucial role, contributing to tumour development, progression, and therapy resistance. Peritoneal metastasis-associated fibroblasts (MAFs) in PSMs exhibit high heterogeneity. Single-cell RNA sequencing technology has revealed that immune-regulatory cancer-associated fibroblasts (iCAFs) seem to be the most prevalent subtype in PSMs. In addition, other major subtypes as myofibroblastic CAFs (myCAFs) and matrix CAFs (mCAFs) were frequently observed across PSMs studies. Peritoneal MAFs are suggested to originate from mesothelial cells, submesothelial fibroblasts, pericytes, endothelial cells, and omental-resident cells. This plasticity and heterogeneity of CAFs contribute to the complex microenvironment in PSMs, impacting treatment responses. Understanding these interactions is crucial for developing targeted and local therapies to improve PSMs patient outcomes.

## Peritoneal surface malignancies

Peritoneal Surface Malignancies (PSMs) were for a long time considered diseases with limited therapeutic options and poor prognosis. Over the last two decades, the combination of perioperative systemic chemotherapy with cytoreductive surgery (CRS) and hyperthermic intraperitoneal chemotherapy (HIPEC) has dramatically improved outcomes [1, 2]. The overall prognosis of PSM is today nearly equivalent to that of patients with metastatic disease at other sites.

The majority of PSMs represent peritoneal metastases that have spread secondarily from a distant primary tumour, and only 3% originate from the peritoneum itself [3]. About 22% derive from colorectal cancer (including appendix), 16% from ovarian cancer, 13% from gastric cancer, 7% from pancreatic cancer, and 9% have an extra-abdominal primary malignancy (mainly breast and lung cancer) [3]. In about one of four patients with peritoneal metastases there are no other clinically apparent sites of metastatic spread [4]. Different types of tumours metastasise to the peritoneum at different rates. The reported relative incidence of peritoneal metastases from ovarian cancer is 60–70%, whereas it is less than 10% for other gynaecological malignancies [5]. Of the gastrointestinal cancers, 43% of patients with gastric cancer and 25% of patients with recurrent colorectal cancer develop metastases that are confined to the peritoneal surface [6, 7]. Pathologic features associated with an increased frequency of peritoneal disease in colorectal cancer are mucinous histology, poor differentiation, pT4 status, nodal metastases and a consensus molecular subtype 4 [4, 8]. The consensus molecular subtype (CMS) is a gene expression-based classification of colorectal cancer, which has been introduced in 2015 [9]. It has contributed to a better understanding of disease heterogeneity and prognosis. CMS4 is the mesenchymal type of colorectal cancer, which is characterised by the activation of transforming growth factor-β, stromal invasion, angiogenesis, and fibrosis. It has been recently identified as the predominant subtype in colorectal cancer-derived peritoneal metastasis [8]. Even though appendiceal cancer is anatomically closely linked to the colon, it shows a different gene expression pattern than colorectal cancer [10]. It metastasises more frequently to the peritoneum, resulting mainly in low-grade appendiceal mucinous neoplasm (LAMN), mucinous appendiceal adenocarcinoma (MACA), and mucinous appendiceal adenocarcinoma of intermediate subtype (MACA-Int) [11]. This suggests that the probability of an individual tumour type forming peritoneal metastases depends very much on the primary site of cancer as well as on its gene expression profile.

**Fig. 1:**
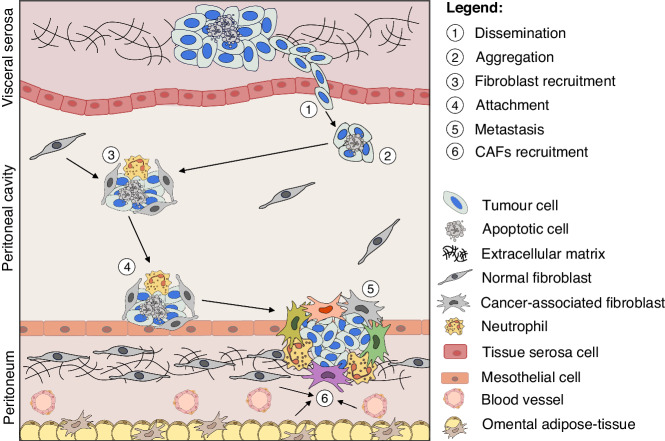
The sequence of events leading to development and establishment of peritoneal carcinomatosis. (1) When the primary tumour overgrows and invades the visceral serosa, cancer cells can be shed into the peritoneal fluid and disseminate across the peritoneal cavity; (2) Tumour cells can enhance their survival by aggregating into multicellular clusters; (3) The increased survival allows the recruitment of immune cells (such as neutrophils) and fibroblast resulting in the formation of heterotypic clusters. (4) Those clusters also display an increased ability to attach to the peritoneum surface (mesothelial cells) and invade into the submesothelial space establishing a peritoneal metastatic tumour (5). Once tumour cell have invaded submesothelial stroma, they must modulate the peritoneal tissue in order to establish a supportive TME. As a consequence, resident fibroblast and other stromal cells are recruited, activated and converted into CAFs (6). The heterogeneity of cancer-associated fibroblasts is represented by the different colours attributed to CAFs.

Although the primary tumours differ considerably in respect to their cellular and genetic background, there are strong similarities in the progression of PSMs resulting from the challenges a tumour cell must overcome to survive and grow in the peritoneal cavity (Fig. 1). Tumour cells must detach from their primary tumour, evade anoikis and gain motility. Once a viable, free cancer cell is floating in the peritoneal cavity, adherence to the peritoneal surface is required to ultimately invade the peritoneum, and form metastases. In order to survive and disseminate better in the peritoneal cavity, metastatic tumours form multicellular clusters. They consist of tumorous as well as non-tumorous cells which aggregate in the peritoneal fluid in a fibrin-mediated manner [12]. Fibroblasts have been frequently found in such ascitic multicellular aggregates in gastric cancer [13], colorectal cancer [14], and ovarian cancer [15]. High-grade serous ovarian cancer cells in the peritoneal fluid are characterised by high expression of integrin α5 which promotes the recruitment of fibroblasts [16]. Furthermore, ovarian cancer cells stimulate their proliferation via transforming growth factor-β1 (TGF-β1) [17]. TGF-β1 was also found in tumour cell-derived exosomes of the malignant ascites which are supposed to additionally enhance peritoneal fibrosis [18]. Fibroblasts which have been recruited, in turn, facilitate the aggregation and compaction of the multicellular clusters by producing collagens and other extracellular matrix (ECM) proteins [17]. They support tumour cell survival in the peritoneal fluid by secreting epidermal growth factor (EGF) and hepatocyte growth factor (HGF) [16, 17]. Moreover, they promote adhesion and invasion of tumour cells by producing matrix metalloproteinase-2 (MMP-2), before becoming essential components of the tumour stroma in newly-formed metastases [16, 17]. Ascitic multicellular aggregates may also contain neutrophils which then form neutrophil extracellular traps (NETs) [19]. Intraperitoneal administration of DNase I in mice showed an 88% decrease in the number of peritoneal metastases confirming that NETs promote survival and adhesion of floating tumour cell aggregates [20].

Although tumour cell aggregates may attach to any intraperitoneal surface, the greater omentum appears to be the preferential site of initial attachment and tumour cells then seed into the rest of the peritoneal cavity [21]. The omentum represents a sheath overlying the abdominal organs which is composed of vascularises adipose tissue embedded with clusters of immune cells termed “milky spots”. Several authors have studied the metastatic pattern of cancer cells after intraperitoneal inoculation in animals and found that these cells preferentially accumulate in the milky spots of the greater omentum as well as the lymphatic lacunae of the diaphragm [21]. This binding may be mediated by the expression of vascular cell adhesion molecule-1 (VCAM-1) and collagen I fibres on the surface of these areas, and of the proangiogenic vascular endothelial growth factor receptor 3 (VEGFR3) by adjacent omental micro vessels [22]. Under healthy conditions, the peritoneum provides a slippery, non-adhesive surface through the microvilli of mesothelial cells, which produce large amounts of proteoglycans and hyaluronic acid, forming the glycocalyx. Ovarian cancer cells express the homing cell adhesion molecule CD44 on their surface, which interacts with hyaluronic acid and mediates intraperitoneal adhesion [23]. CD44 was also found on ovarian cancer-derived exosomes, which transfer this molecule to mesothelial cells, thereby promoting adhesion [24]. It has been shown in colorectal cancer that HGF expressed by associated fibroblasts stimulated tumour adhesion through up-regulation of CD44 via c-Met signalling pathway [25]. Another way in which tumour cell clusters can adhere to the mesothelium is via the tumour antigen CA-125, which is expressed by most tumours that metastasise to the peritoneum. CA-125 binds with high efficiency to mesothelin, a surface protein expressed on normal mesothelial cells lining the peritoneum [26]. This led to the development of mesothelin-targeting agents such as amatuximab and anetumab ravtansine which have been tested in different PSMs in phase I and II clinical trials [27, 28]. Furthermore, intraperitoneal adhesions can also occur as a result of healing processes in the peritoneum. Such processes can be induced by injuries in the course of a surgical procedure, but also as a reaction to the destruction of tissue by tumour growth. Both processes induce the secretion of pro-fibrotic TGF-β, which was found to correlate with the peritoneal adhesion index [29, 30]. Accordingly, high-grade PSMs are usually embedded in fibroblast-rich scar tissues. As both heat and chemotherapy only penetrate a few cell layers deep, such fibrotic PSMs are difficult to target during HIPEC [2]. In summary, it can be concluded that the path from tumour cells spreading into the peritoneal cavity to established surface metastasis is a multi-step process that involves the interplay with non-tumour cells. Fibroblasts appear to play an essential role in this progression.

## Fibroblasts in peritoneal surface malignancies

Already more than 35 years ago, Harold Dorak termed solid tumours as ‘wounds that do not heal’. This is because solid tumours induce a chronic activation of wound healing mechanisms [31]. The destruction of tissue architecture because of persistent growth and invasiveness of tumour cells results in the constant secretion of damage-associated molecular patterns (DAMPs). Furthermore, most cancers show a considerable degree of constant spontaneous cell death, leading to further release of DAMPs [32]. Fibroblasts express pattern recognition receptors (PRRs), such as Toll-like receptors 2 and 4, which sense DAMPs in their surrounding [33]. They migrate to the site of tumour-induced tissue damage, where they are irreversibly activated, and expand to restore the local tissue structures and function [34]. It is well established that these cancer-associated fibroblasts (CAFs) contribute to tumour development, progression and therapeutic resistance through diverse mechanisms. They secrete soluble molecules such as growth factors and chemokines that promote tumour cell growth and inhibit inflammation. Additionally, CAFs provide metabolic substrates for tumour cells, secrete ECM proteins and remodelling enzymes that promote tumour invasion and drive further tissue fibrosis. The different factors and molecular mechanisms involved have already been described in excellent reviews and are, therefore, not discussed here [33, 35–37]. However, it must be pointed out that these review articles describe the significance of CAFs in tumour biology in general. Unfortunately, the special conditions in PSMs are not described in detail, even though CAFs make such an important contribution to PSMs. Most PSMs are characterised by high infiltration of CAFs expressing fibroblast activation protein (FAP), and this is frequently associated with massive fibrosis and scarring [2, 34]. This characteristic can even be used to detect peritoneal metastases by PET/CT scans with [^68^Ga]-labelled FAPI radiotracers [38]. A meta-analysis revealed that ^68^Ga-FAPI PET/CT has a much better sensitivity for the detection of peritoneal metastases than the conventional FDG PET/CT [38].

There are no clearly-defined specific molecular markers to identify CAFs. FAP, α-smooth muscle actin (α-SMA), and platelet-derived growth factor receptor alpha (PDGFRα) are the most commonly used, although their expression is not mandatory [39]. The application of single cell RNA sequencing (scRNA-seq) technology on tumour samples revealed a surprising heterogeneity of CAF functional annotation, even within the same tumour. Dor Lavie and co-authors summarised the results of numerous scRNA-seq studies in a recent review [40]. They grouped the different types of CAFs into three major subsets: myofibroblastic CAFs (myCAFs), immune-regulatory CAFs (iCAFs), and antigen presenting CAFs (apCAFs). myCAFs, also termed ECM-remodelling or wound-healing CAFs, were identified by elevated expression of α-SMA (gene name ACTA2) and show contractile features. iCAFs facilitate tumour immune escape. They secrete cytokines and chemokines, recruit suppressive myeloid and regulatory T cells, exclude cytotoxic lymphocytes and dendritic cells and promote M2 macrophages and type 2 helper T cells. apCAFs express MHC class II-related genes such as CD74, HLA-DRA and HLA-DRB1. It has to be noted that the chosen subclustering into these functional types represents a summary and that individual scRNA-seq studies have made further or different classification. For example, a pan-cancer study by Ma et al. [41], which included primary tumours from liver, colorectal, prostate, breast, ovarian and endometrial origin, appointed five different subtypes: iCAFs, mCAFs (matrix CAFs), meCAFs (metabolic CAFs), pCAFs (proliferating CAFs) and fibroblasts with pericyte/muscle cell properties. In this study, iCAFs and meCAFs are in accordance with Lavie et al. However, the myCAFs defined in Lavie et al. are divided into a) mCAFs (showing ECM remodelling properties via expression of MMP11, CTHRC1 and specific collagens) and b) “real” myCAFs that have contractile properties, expressing mainly ACTA2, MYH11 and RGS5, also resembling pericytes, as mentioned elsewhere [42, 43]. This comparison illustrates the inconsistency of the current literature. There is no consensus on a nomenclature for the different functional subgroups of CAFs. The markers used in the individual studies must be carefully examined when comparing the results. Unfortunately, the number of available PSM-related scRNA-seq data sets is rather limited. At present, only scRNAseq data on ovarian cancer and gastric cancer-derived PSMs are available. Table 1 lists all accessible data sets and indicates the different fibroblast functional subtypes described per study. For further reference, differentially expressed genes between the different reported fibroblast subgroups are shown in Supplementary Table 1. These lists of differential markers are dependent on the context of each study.

**Table 1:** Fibroblast subtypes in peritoneal surface malignancies.

Primary disease	Tumour stages	Type	Fibroblast types	Reference
Ovarian cancer-derived PSM				
HGSOC (*n* = 3) LGSOC (*n* = 1)	IIIA (*n* = 1), IIIB (*n* = 1), IIIC (*n* = 2)	Solid	iCAF	[44]
HGSOC	NA	Ascites	iCAF, non-iCAF	[45]
HGSOC (*n* = 1) LGSOC (*n* = 1)	NA	Solid	myCAF, mCAF	[53]
HGSOC	NA	Ascites	iCAF, myCAF	[46]
NA	NA	Solid	paCAF, iCAF, myCAF	[116]
HGSOC	IIIB (*n* = 1), IIIC (*n* = 2)	Solid, ascites	mCAF, iCAF, myCAF, STAR^+^ CAF	[47]
HGSOC	Late stage HGSOC	Ascites	mCAF	[55]
HGSOC	IIIC (*n* = 3), IVA (*n* = 7), IVB (*n* = 1)	Solid	mCAF, iCAF, myCAF	[50]
High-grade serous tubo-ovarian cancer	IIIC (*n* = 2), IVB (*n* = 4)	Solid	myCAF, mCAF, iCAF	[51]
HGSOC	IIC, IIIC (*n* = 1 of each type)	Solid	STAR^+^ CAF, myCAF, apCAF, TNF-related (iCAF-like)	[52]
Gastric cancer-derived PSM				
Gastric adenocarcinoma	Intestinal (based on Lauren’s classif.)	Solid	iCAF, myCAF	[56]
Gastric adenocarcinoma	Diffuse (*n* = 20), Intestinal (*n* = 2) (based on Lauren’s classif.)	Ascites	iCAF / iCAF-like, mCAF	[57]

The number of patients per tumour stage is shown in parentheses. The definition of the fibroblast types followed the direct definitions provided by the authors, where applicable. In case of absence of a definition, we defined CAF subtypes following definitions provided by Ma et al. and Lavie et al. [40, 41]. vCAF/pericytes were regarded as myCAF.

*HGSOC* High-grade serous ovarian cancer, *LGSOC* Low-grade serous ovarian cancer, *myCAF* myofibroblastic CAF, *iCAF* immune-regulatory CAF, *mCAF* matrix CAF, *paCAF* perpetually activated CAF, *apCAF* antigen presenting CAF, *NA* non applicable

### Ovarian cancer-derived PSMs

In a study on solid ovarian cancer samples, peritoneal metastasis-associated fibroblasts and fibroblasts from primary tumour expressed increased levels of collagen and ECM-remodelling proteins (MMP2/11), when compared to fibroblasts from normal adjacent tissue [44]. However, when fibroblasts from the primary cancer were compared to those from peritoneal metastases, the latter expressed a significant number of markers associated with iCAFs. This is in accordance with another study on high-grade serous ovarian cancer (HGSOC), which also found a prevalence of iCAFs in ascites, expressing IL-6, C1Q and CXCL1/2/10/12, among other markers [45]. A second CAF type in this study was defined as non-iCAFs. Carvalho et al. used the same scRNAseq cohort and confirmed the previous findings, additionally annotating the non-iCAF functional type as myCAFs [46]. In a study from Loret et al. on ascites and solid peritoneal metastases from HGSOC, four different CAF subtypes were identified (myCAF, iCAF, mCAF, STAR^+^ CAF). iCAFs were the prevalent type in ascites, whereas they were second prevalent in solid metastases, preceded by mCAFs. Interestingly, iCAFs were absent from primary tumours in treatment-naïve patients. After carboplatin-paclitaxel treatment, mCAFs and myCAFs showed increased expression of CXCL12 and IL-6, respectively, thus acquiring more iCAF-related properties [47]. Expression of these ligands could alter their communication with other cell types in the TME in an unfavourable manner [48, 49]. Zhang and co-workers divided HGSOC PSM samples based on the expression of a stress-response signature in stress-high and stress-low cancers [50]. Of note, stress-high cancers were related to poor prognosis, and only iCAFs were significantly enriched in stress-high samples. iCAF-like cells were also detected in another HGSOC study [51] which used the subtyping system of Qian et al. to assign CAF subtypes [43]. Notably, a subtype that was detected only in normal adjacent and metastatic omentum (FB_CALB2) was described to have mesothelial origin and expression of iCAF-related genes, including IL-6, IL-18, COL8A1, CXCL16, CCL2, CXCL1 and IL6ST. Other CAF types related to poor overall survival were: (a) mCAF-like cells (defined as FB_COMP) which were present equally in ovarian primary tumours, omental metastases and peritoneal metastases; and b) myCAF-like cells (defined as FB_MYH11), which were mostly present in normal ovarian tissue. Deng et al. identified four different types of fibroblasts in solid HGSOC samples: mCAF, STAR+, apCAF and TNF-related. However, STAR+, and TNF CAFs showed expression of immune-related factors and, combined with the de facto immunomodulatory apCAFs, formed a “supergroup” of immunoregulatory, iCAF-like cells. This group was the most prevalent in peritoneal metastasis [52]. Only few studies demonstrated subtypes other than iCAFs as the main subtype in ovarian cancer-related PSMs. PSM solid samples HGSOC and Low-grade SOC (LGSOC) [53] showed mainly a CAF type related to angiogenesis and evasion of apoptosis, related to vascular CAFs (vCAFs) and pericytes (defined here [54]), whereas primary tumours from the same study mostly contained matrix CAFs (mCAFs) (defined here [40]). mCAFs were also the main subtype identified in ascites of five HGSOC patients [55], showing proEMT properties.

### Gastric cancer-derived PSMs

In a study on multiple metastasis locations from gastric adenocarcinoma, iCAFs were the main CAF type in PSMs, and the CXCL12-CXCR4 module was the main route of communication of iCAFs with several target cell types, including T cells, B cells, endothelial, dendritic cells, neutrophils and macrophages [56]. On the other hand, mCAF fibroblasts were the most prevalent in gastric cancer ascites samples [57]. Presence of the mCAF signature showed increased risk of metastasis and/or recurrence.

**Fig. 2:**
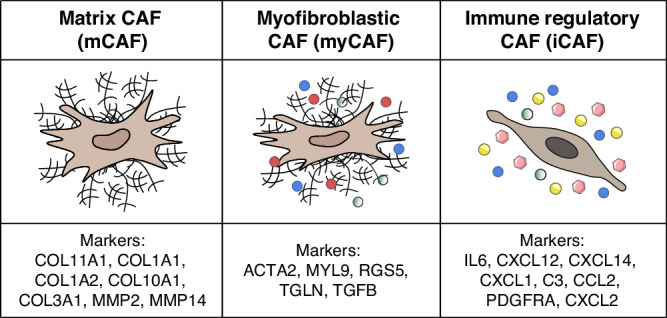
The most commonly found fibroblast subtypes in peritoneal surface malignancies. In case a specific definition by the authors of the studies was missing, reported markers from each study were evaluated according to markers from Ma et al., Lavie et al. and Cords et al., in order to define the functional subtypes of the reported fibroblast groups. The most frequently reported definition markers per functional subtype among the PSM scRNAseq studies are shown [40, 41, 54]. myCAFs and mCAFs, modulate the extracellular matrix and generate a stiff tumour microenvironment, favouring tumour metastasis and chemoresistance. However, only myCAF express in addition ACTA2 and show a contractile phenotype. iCAF-secreted factors can induce inflammatory pathways, but they have also been reported to attract immunosuppressive regulatory T cells and promote anti-inflammatory polarisation of myeloid cells.

In summary, despite all the differences between the individual studies, there is a clear prevalence of three functional CAF subtypes in PSM: mCAFs, myCAFs, and iCAFs (Fig. 2). Notably, iCAFs or fibroblasts presenting iCAF-related traits (iCAF-like) are the dominant fibroblast subtype in the majority of studies of ovarian cancer-derived PSMs. In addition, in a subset of studies, iCAFs were exclusively present in the PSM tumour microenvironment, and not in primary samples [44, 47, 50]. This finding could suggest an enrichment of the iCAF functional subtype in PSM, possibly related to immunoregulatory functions and adverse prognosis. In some studies, the prevalent peritoneal MAF subsets are not iCAFs, but rather myCAFs or mCAFs. This discrepancy could be eventually related to different clinical parameters, including disease subtype, tumour stages etc. The total number of patients in current studies is insufficient to correlate such variations with clinically relevant variables, highlighting the need for new, extensive cohorts with more patients in the scRNA-seq analysis of fibroblasts in ovarian cancer-derived PSMs. This limitation is even more evident in the other PSM types. Only two studies provide information about peritoneal MAFs in metastatic gastric cancer. Specifically, in gastric cancer, one study defined iCAFs as the main type, whereas the other defined mCAFs. An important difference between these cohorts is the cancer subtype (intestinal vs diffuse, respectively). Notably, scRNA-seq studies on fibroblasts from colorectal cancer-derived PSMs are absent.

Overall, iCAFs are the main subtype metastasis-associated fibroblasts (MAF) in PSMs, and certain studies claimed an enrichment of the IL-6/JAK/STAT pathway. This is in contrast to primary and metastatic cancers in other locations (pancreas, breast, lung, liver), where myCAFs and mCAFs are the dominant fibroblast types [40]. This difference could reflect the importance of the local microenvironment. Considering the plasticity of fibroblasts, it would be possible that the local microenvironment shapes the MAFs and vice versa. It should be noted that scRNAseq data only provides information about which cell populations are present in a tissue at a certain point in time. However, no conclusions can be drawn as to what led to this cell composition. In order to find out what could have led to the formation of MAFs, one must refer to other studies.

### Origin of peritoneal metastasis-associated fibroblasts

**Fig. 3:**
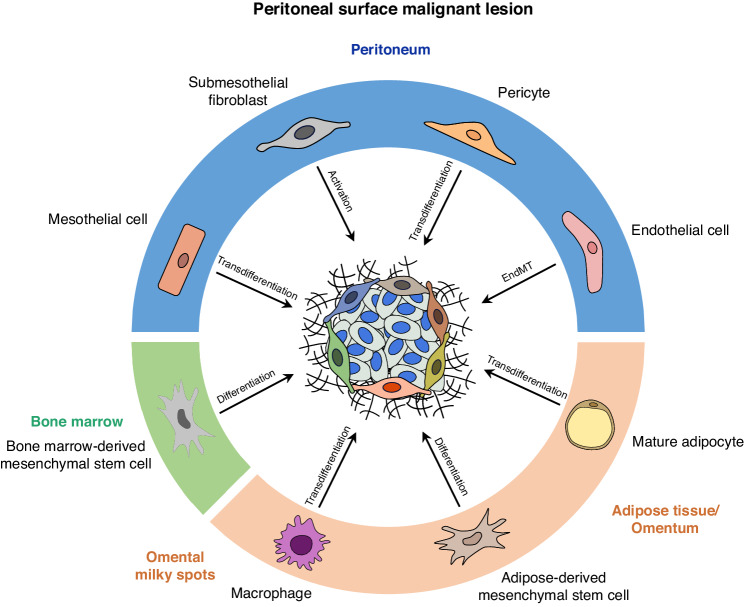
The multi-cellular origins of cancer-associated fibroblasts (CAFs) in peritoneal surface malignancies. Signals from the tumour microenvironment can activate precursor cells from different tissue sources: peritoneum, adipose tissue/omentum, and bone marrow. The potential sources include mesothelial cells, submesothelial fibroblasts, pericytes, endothelial cells, mature adipocytes, adipose-derived mesenchymal stem cell, macrophages, and bone marrow-derived mesenchymal stem cells. The process which results from the conversion of those cellular precursors is indicated by the arrows. The diverse sources of CAFs lead to the emergence of diverse CAF subpopulations and heterogeneity within the tumour microenvironment.

The plasticity and heterogeneity of fibroblast phenotypes and functions are supposed to be primarily related to their diverse cellular origins [40]. Using mouse models, it was demonstrated that the majority of CAFs derived from local rather than from circulating precursors [58]. Thus, the source of stromal fibroblasts depends on the specific environment surrounding malignant cells. In PSMs this is the peritoneum or the greater omentum. Structurally, the peritoneum comprises the mesothelium, a basal lamina consisting of ECM, and the submesothelial stroma [38]. The latter include submesothelial fibroblasts, muscle cells, and finally endothelial cells and pericytes of the microvasculature. The omentum represents an adipose tissue with immune cell aggregates in the milky spots. Various cell types of the peritoneum or of the greater omentum have the capacity to serve as a source of peritoneal MAFs (Fig. 3 and Table 2).

**Table 2:** Possible cellular origin sources contributing to accumulation of activated fibroblasts in peritoneal fibrosis and cancer-associated fibroblasts within the tumour microenvironment.

Cell of origin	Process	Mechanism	Comment	Reference
**Malignant**				
*Ovarian Cancer*				
MC	MMT	TGF-β1	Analysis of biopsies from OV patients with peritoneal metastasis	[62]
MC	MMT	TGF-β1 -> ↑ VEGF	A novel mechanism of neovascularization in peritoneal dissemination	[117]
MC	MMT	TGF-β1 - Smad3	Ascitic fluid-isolated MCs	[67]
O-NF	Activation	TGF-β1 - Smad2	3D culture model and an in vivo assay	[80]
NF	Activation	TC-derived exosomal miR-141 - > YAP1/CXCL1/CXCRs	Mouse primary stromal fibroblasts	[118]
O-MA	Transdiff.	Wnt/β-catenin -> TGF-β1	Dedifferentiated adipocytes support proliferation and migration of OV cells	[119]
AD-MSC	Diff.	TGF-β1 + Rac1/PAK1 signalling	CAF-like differentiation induced by tumour cells could happen before tumour implantation	[101]
AD-MSC	Diff.	TC-derived exosomes -> TGF-β	Tumour-derived exosomes induced the myofibroblastic phenotype	[120]
AD-MSC	Diff.	TC-derived exosomes -> TGF-β	Different tumour cells may specifically exert their own biological effects	[121]
AD-MSC	Diff.	-	Elevated the expression of MMP2 and MMP9	[100]
AD-MSC	Diff.	LPA - > TGF-β1/Smad	Malignant Ascites Induce the differentiation	[102]
*Gastric cancer*				
MC	MMT	Observational study	FAP expression associated with peritoneal dissemination and poor prognosis	[65]
MC	MMT	TGF-β1 - Smad2	MMT promotes peritoneal carcinomatosis	[63]
MC	MMT	TGF-β1	Tumours contained activated MC-derived fibrous tissue	[69]
MC	MMT	TGF-β/Smad	Tranilast (drug of low toxicity) inhibits the effects of TGF-β-induced MMT	[122]
MC	MMT	Tks5	MCs create invasion front by guiding cancer cells, scirrhous gastric carcinoma	[64]
Pericyte	Transdiff.	BMP transfer (exosomes) -> PI3K/AKT + MEK/ERK	Tumour cells induced the transition of pericytes to CAFs by exosomes-mediated BMP transfer	[83]
O-MA	Transdiff.	TGF-β1/Smad		[93]
Peritoneal MSC	Diff.	TGF-β1	Peritoneal fluid cells engrafted in metastatic nodules and were mainly located at the fibrous area	[98]
BM-MSC	Diff.	CXCL12 + TGF-β1 + DNA hypomethylation	20% of all CAFs found within the TME	[111]
BM-MSC	Diff.	Observational study	Bone marrow-derived cells following allogeneic stem cell transplantation	[112]
FC	Diff.	CXCL12/CXCR4 + TGF-β1	-	[107]
MSC	Diff.	-	GC-MSCs-primed neutrophils could induce the differentiation of MSC into CAFs	[123]
MSC	Diff.	TGF-β/Smad	CG exosomes induces differentiation of umbilical cord-derived MSC into CAFs	[124]
*Colorectal cancer*				
MC	MMT	Observational study	MMT is a key event in the early stages of peritoneal dissemination	[66]
MC	MMT	Observational study	Presence of mesothelial markers in CAFs of CRC peritoneal metastases	[70]
MC	MMT	TGF-β1	Analysis of biopsies from CRC patients with peritoneal metastasis	[62]
SM-F	Activation	-	Gene signature associated with peritoneal invasion	[125]
EC	EndMT	TGF-β1 -> ↑ tubulin-β3	Microtubules enriched in tubulin-β3	[88]
BM-MSC	Diff.	GRP78 -> TGF-β/Smad	GRP78 is secreted by tumour cells	[126]
BM-MSC	Diff.	Notch–Jagged1 + TGF-β/Smad	Direct contact induces the differentiation	[127]
*Endometrial cancer*				
MC	MMT	TGF-β1	Analysis of biopsies from endometrial cancer patients with peritoneal metastasis	[62]
O-ASC	Diff.	-	Comparison to other subcutaneous adipose-derived mesenchymal stem cells	[128]
O-ASC	Diff.	-	tumorigenic hybrids spontaneously formed between human O-ASCs and endometrial cancer cells	[129]
*Pancreatic cancer*				
MC	MMT	TGF-β1	Analysis of biopsies from pancreatic cancer patients with peritoneal metastasis	[62]
MA	Transdiff.	WNT5a	Treatment with WNT5a neutralising antibody completely reverted the process	[130]
BM-MSC	Diff.		Sustained expression of CXCL12	[110]
*Pan-cancer*				
Peritoneal macrophages	Transdiff.	TGF-β1 + cell-matrix adhesion	Malignant ascites from tumour dissemination of digestive organ cancers	[105]
**Non-malignant**				
*Peritoneal dialysis*				
MC	MMT	TGF-β1	Exogenous BMP4 or IGFBP4 ameliorates TGF-β1-induced MMT	[131]
MC	MMT	TGF-β1 -> hyperglycolysis	Inhibition of hyperglycolysis in mesothelial cells prevents peritoneal fibrosis	[132]
MC	MMT	↓ BMP-7 + ↑ TGF-β1	rh-BMP-7 blocks TGF-β1-induced MMT of MC	[133]
MC	MMT	TGF-β1	Morphologic and functional characterisation	[134]
MC	MMT	↓ miR-15a-5p/VEGFA -> TGF-β1/Smad2	miR-15a-5p suppresses inflammation and fibrosis	[135]
MC	MMT	TGF-β1/Smad	Loss of JLP promotes PD-related peritoneal fibrosis	[136]
MC	MMT	TGF-β1 + IL-1β -> ↑ Snail -> α2 integrin	Mesothelial cells also acquire a migratory, invasive and fibrogenic phenotype	[59]
MC	MMT	Observational study	MMT is an early event during peritoneal dialysis	[137]
MC	MMT	TGF-β1 + IL-1β -> ↑ VEGF	MMT-activated MC secrete VEGF -> high peritoneal transport rate	[138]
MC	MMT	ERK/NF-κB/Snail1 pathway	MCs treated with effluent from patients undergoing peritoneal dialysis	[139]
MC + SM-F	MMT + Activation	TGF-β1 + IL-1β -> ↓ CD34	Loss of CD34+ stromal cells	[72]
MC + EC	MMT + EndMT	TGF-β1 - > MEK‐ERK1/2‐Snail‐1	Caveolin‐1 as an important regulator of MMT/EndMT	[90]
MC + FC + EC	MMT + Diff. + EndMT	TGF-β1	Blocking TGF-β1 protects peritoneal fibrosis	[140]
SM-F	Activation	TGF-β1	Lineage tracing study shows SM-F as the major myofibroblast precursors in peritoneal fibrosis	[75]
Pericyte	Transdiff.	GSDMD/IL-1β pathway	Induced by NLRP3 inflammasome activated macrophages	[84]
*Peritoneal fibrosing syndrome*				
SM-F	Activation	-	Selective depletion of FSP1+ fibroblasts prevent fibrosis	[141]
*Peritoneal inflammation*				
Peritoneal macrophages	Transdiff.	-	Peritoneal macrophage transdifferentiation into tissue capsule myofibroblasts	[142]
Peritoneal macrophages	Transdiff.	TGF-β1	Inhibition of TGF-β1 signalling pathway supressed the transdifferentiation	[143]

*MC* mesothelial cell, *SM-F* submesothelial fibroblast, *EC* endothelial cell, *FC* fibrocyte, *O-NF* omental-normal fibroblast, *NF* normal fibroblast, O-MA omental mature adipocyte, AD-MSC adipose-derived mesenchymal stem cell, *MSC* mesenchymal stem cell, *BM-MSC* bone marrow-derived mesenchymal stem cell, *O-ASC* omental adipose-derived stromal cell, *MMT* mesothelial-mesenchymal transition, *EndMT* endothelial-mesenchymal transition, *TGF-β1* transforming growth factor beta 1, *BMP* bone morphogenetic protein, *miR* microRNA, *VEGF* vascular endothelial growth factor, *SMAD* mothers against decapentaplegic, *IL-1β* interleukin 1 beta, *ERK* extracellular signal-regulated kinase, *NF-κB* factor nuclear kappa B, *MEK* mitogen-activated protein kinase, *GSDMD* gasdermin D, *YAP1* yes-associated protein 1, *CXCL* chemokine (C-X-C motif) ligand, *CXCR* CXC chemokine receptor, *Rac1* Rac family small GTPase 1, *PAK1* P21 (RAC1) activated kinase 1, *TC* tumour cell, *LPA* lysophosphatidic acid, *PI3K* phosphoinositide 3-kinase, *DNA* Deoxyribonucleic acid, *GRP78* glucose-regulated protein 78, *IGFBP* insulin like growth factor binding protein, *rh* recombinant human protein, *JLP* JNK-associated leucine zipper protein, *FSP1* ferroptosis suppressor protein 1, *NLRP3* NLR family pyrin domain containing 3, *3D* three-dimensional, *CAF* cancer-associated fibroblast, *MMP* matrix metalloproteinase, *FAP* fibroblast activation protein, *TME* tumour microenvironment, *Diff.* differentiation, *Transdiff.* Transdifferentiation.

#### Mesothelial cells

Mesothelial cells, which are specialised squamous epithelial cells, can transdifferentiate from mesothelial to mesenchymal states. The first evidence came from a study in peritoneal dialysis patients [59]. Indeed, later studies confirmed that similar transdifferentiation takes also place in PSMs of ovarian, endometrial and colorectal cancer patients [60–62]. The mesothelial-to-mesenchymal transition (MMT) process is characterised by a decrease of epithelial markers and increase in mesenchymal markers [59, 62]. Over time, there is a general downregulation of cytokeratins that are replaced by vimentin, resulting in loss of the cuboid epithelial morphology and acquisition of a spindle-shaped phenotype. Those fibroblast-like mesothelial cells lose contact inhibition, proliferate in a non-structured manner and acquire a migratory phenotype. The TGF-β1/Smad axis seems to be critical for full induction of this phenotype [63]. Sandoval et al. observed that peritoneal MAFs from biopsies of ovarian cancer co-expressed CAF-specific marker α-SMA alongside with the mesothelial markers cytokeratin and calretinin [62]. Similarly, in scirrhous gastric carcinoma, invading lead cells exhibited partial overlap in α-SMA and calretinin [64]. Miao et al. showed in gastric cancer that mesothelial expression of FAP was associated with poor prognosis, higher TNM stage and incidence of peritoneal dissemination by promoting cancer cells chemotaxis and adhesion [65]. During the colonisation of the peritoneum by colonic tumour cells, it was shown that CAFs found in the advanced primary tumours arise from MMT of mesothelial cells covering the visceral serosa [66]. Migrating mesothelial cells with spindle-like morphology were observed in the submesothelial region. These cells were negative for mesenchymal markers observed in invasive neoplasms. This resembles a transitional state of mesothelial cells, suggesting that MMT process is a critical event happening at the early stages of peritoneal dissemination. Interestingly, the in vitro treatment of omental mesothelial cells either with conditioned media of ovarian or colorectal cancer cells resulted in acquisition of MMT phenotypic features [62]. In a similar way, ex vivo cultures of mesothelial cells isolated from ascites of ovarian cancer patients undergo MMT and promote tumour growth in a xenograft mouse model [67]. Likewise, in cancer xenograft models the simultaneous injection of human mesothelial cells and tumour cells increased tumour fibrosis and peritoneal dissemination of gastric and ovarian cancer [68, 69]. Demuytere et al. suggested that MMT appears to be an important source of CAFs in the TME of CRC peritoneal metastases [70]. The immunohistostaining of biopsies showed overlap of mesothelial and CAFs markers. Recently, it was demonstrated in a pancreatic cancer model, that mesothelial cells are the cellular origin of antigen-presenting CAFs. By employing linage tracing methods Huang and colleagues identified the mesothelial-derived apCAFs as the main inducers of Treg formation in PDAC [71]. Interestingly, the treatment with mesothelin antibody inhibited the transdifferentiation. All together, these findings strengthen the concept that the activation of MCs through MMT process is a presumable source of peritoneal MAFs in PSMs.

#### Submesothelial fibroblasts

Peritoneal MAFs can also arise from resident submesothelial fibroblasts. Under normal conditions, fibroblasts in the peritoneum only occur in the submesothelial connective tissue [38]. Their CD34 expression suggests that they originate from blood-borne fibrocytes [72]. The absence of α-SMA, desmin, or FAP indicate that they are in a quiescent state. The specific conditions in the microenvironment of the peritoneal metastasis, such as tissue destruction, hypoxia and starvation, lead to attraction, activation and epigenetic reprogramming of the resident submesothelial fibroblasts, resulting in a constantly activated phenotype [73, 74]. Chen et al. by applying lineage tracing methods, identified the SM fibroblasts as the major precursors of myofibroblasts in peritoneal fibrosis [75]. In a similar approach, it was shown that around 25% of ACTA2^+^ CAFs were generated via activation of resting fibroblasts in a gastrointestinal cancer model [76]. Tumour cell secretion of TGF-β family members facilitates the activation and recruitment of resident fibroblasts in a Src- and Smad-dependent manner, respectively [77, 78]. Resident fibroblasts in the metastatic niche might be pre-activated even before the tumour cells arrive at the distant site. In prostate cancer, the activation of local fibroblasts in the pre-metastatic niche, similarly to those in the primary tumour, accelerates and supports the growth of metastatic colonies [79]. Those activated fibroblasts increase the expression of ECM proteins and chemokines. Cai et al. reported that activated normal omentum fibroblasts behave upon TGF-β1 treatment similarly to CAFs isolated from metastatic ovarian cancer patients, and those cells could also be found in omentum of these patients without metastasis [80]. Activation of omental normal fibroblast into CAFs promote the peritoneal metastasis of ovarian cancer [81].

#### Pericytes

Pericytes are mural cells involved in the microcirculation. It has been observed in a xenograft mouse model that platelet-derived growth factor-BB (PDGF-BB), which is overexpressed in several tumour types, induces a pericyte-to-fibroblast transition [82]. Inhibition of the PDGF-BB-PDGFRβ signalling reduced this transition as well as tumour invasion and metastasis. A study on gastric cancer-derived exosomes showed that they are able to induce the transdifferentiation of pericytes into CAFs by an PI3K/AKT and MEK/ERK pathway-dependent mechanism [83]. Although both AKT, as well as ERK, are responsive to PDGF, the potential role of this factor was, unfortunately, not investigated. Transition from pericytes to fibroblasts was also observed in a peritoneal dialysis model [84]. Macrophages were found to induce the transformation via the gasdermin/IL-1β axis. Recently, a single-cell RNA sequencing study showed the contribution of pericytes in human kidney fibrosis [85]. This observation supports the previously described origin of myofibroblasts in fibrosis by a lineage tracing study [86]. Thus, the transdifferentiation of pericytes into fibroblasts seems to be a general response and not restricted to malignancies.

#### Endothelial cells

It was shown in a spontaneous pancreatic carcinoma model that TGF-β1 induces endothelial cells to undergo a phenotypic conversion into fibroblast-like cells [87]. This was associated with the emergence of mesenchymal marker fibroblast-specific protein-1 (FSP1) and down-regulation of CD31/PECAM. Invasive colon cancer cells were found to potentiate the formation of endothelial cell-derived fibroblasts via the TGF- β1/tubulin-β3 axis [88]. Lineage tracing studies demonstrated that endothelial cell-to-fibroblast transdifferentiation is also involved in kidney and peritoneal fibrosis of peritoneal dialysis patients [89, 90].

#### Omental cells

The omentum, which consists of well-vascularises and innervated layer of adipose tissue, is a common place of peritoneal metastasis. Omental adipocytes can promote peritoneal dissemination of PSMs via their transdifferentiation into fibroblasts [91]. The ability of cancer cells to induce such transdifferentiation was found for gastric cancer and ovarian cancer [92, 93]. Tumour cell-derived soluble factors, such as TGFβ1 inhibit adipogenesis of adipose-derived mesenchymal stem cells (AD-MSCs) while increasing the fibroblastic differentiation followed by ECM deposition [94]. The greater omentum is known to contain abundant AD-MSCs [95]. It was reported that AD-MSCs migrate towards tumour engraftments in animal models [96]. In a xenograft model, combination of lineage tracing with scRNA-seq analysis revealed that AD-MSCs can differentiate into diverse CAFs populations [97]. In an in vivo study, Kitayama et al. have demonstrated that MSC-like cells found in peritoneal fluid of PSM patients play an important pro-metastatic role [98]. Stimulation of AD-MSCs with TGF-β induced their differentiation to a fibroblast-like phenotype expressing type I collagen, vimentin, α-SMA and FAP. This was associated with an enhanced rate of metastasis formation. Human AD-MSCs isolated from omentum of ovarian cancer patients promote survival and chemoresistance of tumour cells in vitro [99]. Tang et al. and others have demonstrated the contribution of AD-MSCs to the peritoneal MAF generation and their support of peritoneal dissemination [100, 101]. It was found that ascites of ovarian cancer patients are enriched in lysophosphatidic acid. This bioactive phospholipid stimulates the differentiation of AD-MSCs into CAFs via TGF-b1/smad axis [102]. Furthermore, it was shown in an ovarian cancer mouse model that adipose-derived α-SMA^+^ stroma is the preferred engraftment location for peritoneal metastasis formation [103].

#### Macrophages

The omental milky spots increase in size and number in response to the initial colonisation of tumour cells, mainly by recruitment of macrophages which acquire an anti-inflammatory polarisation and suppress anti-tumour immune activities [104]. It was observed that macrophages from malignant ascites of gastrointestinal cancer patients transdifferentiate into fibroblasts though activation of TGF-β signalling and cell-matrix adhesion [105]. The authors found that in an animal model of PSM, these cells promote tumour growth. A scRNA-seq analysis to unveil omentum activation processes showed the existence of a novel population which co-expressed both fibroblast and macrophage markers, suggesting the presence of macrophage-derived fibroblast cells [106].

Not only cells of the peritoneal cavity but also cells from other sites of the body are potential sources of peritoneal MAFs. For example, a study in gastric cancer revealed that bone marrow-derived fibrocytes are recruited from the peripheral blood into the TME where they support tumour growth and fibrosis by differentiating into fibroblasts [107]. Cancer patients were found to have higher numbers of fibrocytes and bone marrow-derived MSCs (BM-MSCs) in their peripheral blood [108]. Evidence arising from tracing studies suggests that BM-MSCs can also be a source of fibroblasts [74, 109]. Following exposure to TGF-β, BM-MSCs show DNA hypomethylation and their gene expression shifts towards myCAF phenotype signature. Mishra et al. have shown that exposure of BM-MSCs to tumour conditioned media increased expression of α-SMA and FSP1 simultaneously with sustained expression of CXCL12, inducing their differentiation into a CAF-like population [110]. In a study on gastric cancer, Quante et al. utilised GFP-labelled BM-MSCs to illustrate that MSCs are recruited to tumour site in a TGF-β- and CXCL12-dependent manner, accounting for around 20% of all CAFs found within the tumour mass [111]. Supporting these observations, Worthley et al. reported that female patients with gastrointestinal neoplasias, who received BM transplants from male donors, presented Y chromosome-positive CAFs within the TME [112]. Finally, some studies even propose that malignant cells or tumour stem cells might also account for the CAF pool through the EMT process [113, 114]. However, earlier data suggest that genetic mutations present in both cancer cells and CAFs are mostly mutually exclusive [115].

### Conclusion and outlook

The spontaneous death of disseminating tumour cells and the tumour-induced tissue destruction at the adhesion site trigger a chronic tissue repair reaction in which various types of fibroblasts are involved. They are recruited from various cell sources and attempt to heal the wound and restore the homoeostasis. However, in doing so, they support the survival, adhesion, growth and spread of tumour cells and make them more resistant to chemotherapy. Although this role of fibroblasts can also be observed in many other types of cancer, it is of particular importance in PSM. Firstly, they exhibit strong fibroblastic infiltrates. Perhaps more importantly, the site of spread and adhesion is more accessible than in other metastatic malignant tumours. A better understanding of the origin of peritoneal MAFs and their interplay with tumour and immune cells may help to identify specific drug targets. This could contribute to the development of new drugs that complement the current treatment options.

### Supplementary information

Supplementary Table 1

## References

[1] Cortés-Guiral D, Hübner M, Alyami M, Bhatt A, Ceelen W, Glehen O. et al. Primary and metastatic peritoneal surface malignancies. Nat Rev Dis Primers. 2021;7:91. http://www.ncbi.nlm.nih.gov/pubmed/34916522. 34916522 10.1038/s41572-021-00326-6

[2] Sugarbaker PH. A narrative review of what can HIPEC do. Eur J Surg Oncol. 2023;49:106976 http://www.ncbi.nlm.nih.gov/pubmed/37453879. 37453879 10.1016/j.ejso.2023.07.002

[3] Solon JG, O’Neill M, Chang KH, Deady S, Cahill R, Moran B, et al. An 18 year population-based study on site of origin and outcome of patients with peritoneal malignancy in Ireland. Eur J Surg Oncol. 2017;43:1924–31. http://www.ncbi.nlm.nih.gov/pubmed/28583791. 28583791 10.1016/j.ejso.2017.05.010

[4] Carr NJ. New insights in the pathology of peritoneal surface malignancy. J Gastrointest Oncol. 2021;12:S216–29. http://www.ncbi.nlm.nih.gov/pubmed/33968439. 33968439 10.21037/jgo-2020-01 PMC8100698

[5] Burg L, Timmermans M, van der Aa M, Boll D, Rovers K, de Hingh I, et al. Incidence and predictors of peritoneal metastases of gynecological origin: a population-based study in the Netherlands. J Gynecol Oncol. 2020;31:e58. 10.3802/jgo.2020.31.e58 PMC7440978 32808491

[6] Rijken A, Lurvink RJ, Luyer MDP, Nieuwenhuijzen GAP, van Erning FN, van Sandick JW, et al. The burden of peritoneal metastases from gastric cancer: a systematic review on the incidence, risk factors and survival. J Clin Med. 2021;10:4882. 10.3390/jcm10214882 PMC8584453 34768402

[7] Segelman J, Granath F, Holm T, Machado M, Mahteme H, Martling A. Incidence, prevalence and risk factors for peritoneal carcinomatosis from colorectal cancer. Br J Surg. 2012;99:699–705. http://www.ncbi.nlm.nih.gov/pubmed/22287157. 22287157 10.1002/bjs.8679

[8] Lenos KJ, Bach S, Ferreira Moreno L, ten Hoorn S, Sluiter NR, Bootsma S, et al. Molecular characterization of colorectal cancer related peritoneal metastatic disease. Nat Commun. 2022;13:1–14. https://www.nature.com/articles/s41467-022-32198-z. 35927254 10.1038/s41467-022-32198-z PMC9352687

[9] Guinney J, Dienstmann R, Wang X, De Reyniès A, Schlicker A, Soneson C, et al. The consensus molecular subtypes of colorectal cancer. Nat Med. 2015;21:1350–6. 10.1038/nm.3967. 26457759 10.1038/nm.3967 PMC4636487

[10] Levine EA, Blazer DG, Kim MK, Shen P, Stewart JH, Guy C, et al. Gene expression profiling of peritoneal metastases from appendiceal and colon cancer demonstrates unique biologic signatures and predicts patient outcomes. J Am Coll Surg. 2012;214:599–606. http://www.ncbi.nlm.nih.gov/pubmed/22342786. 22342786 10.1016/j.jamcollsurg.2011.12.028 PMC3768122

[11] Sugarbaker PH, Chang D, Liang J. Pathogenesis of histologic variations of appendiceal mucinous neoplasms. Eur J Surg Oncol. 2023;49:895–901. http://www.ncbi.nlm.nih.gov/pubmed/36863914. 36863914 10.1016/j.ejso.2023.02.014

[12] Miyazaki M, Nakabo A, Nagano Y, Nagamura Y, Yanagihara K, Ohki R, et al. Tissue factor-induced fibrinogenesis mediates cancer cell clustering and multiclonal peritoneal metastasis. Cancer Lett. 2023;553:215983. https://linkinghub.elsevier.com/retrieve/pii/S0304383522004700. 36404569 10.1016/j.canlet.2022.215983

[13] Yasuda T, Koiwa M, Yonemura A, Miyake K, Kariya R, Kubota S, et al. Inflammation-driven senescence-associated secretory phenotype in cancer-associated fibroblasts enhances peritoneal dissemination. Cell Rep. 2021;34:108779 http://www.ncbi.nlm.nih.gov/pubmed/33626356. 33626356 10.1016/j.celrep.2021.108779

[14] Poonpanichakul T, Shiao MSS, Jiravejchakul N, Matangkasombut P, Sirachainan E, Charoensawan V, et al. Capturing tumour heterogeneity in pre- And post-chemotherapy colorectal cancer ascites-derived cells using single-cell RNA-sequencing. Biosci Rep. 2021;41:20212093. http://www.ncbi.nlm.nih.gov/pubmed/34708245. 10.1042/BSR20212093 PMC8655500 34708245

[15] Wintzell M, Hjerpe E, Åvall Lundqvist E, Shoshan M. Protein markers of cancer-associated fibroblasts and tumor-initiating cells reveal subpopulations in freshly isolated ovarian cancer ascites. BMC Cancer. 2012;12:359. http://www.ncbi.nlm.nih.gov/pubmed/22901285. 22901285 10.1186/1471-2407-12-359 PMC3517779

[16] Gao Q, Yang Z, Xu S, Li X, Yang X, Jin P, et al. Heterotypic CAF-tumor spheroids promote early peritoneal metastatis of ovarian cancer. J Exp Med. 2019;216:688–703. http://www.ncbi.nlm.nih.gov/pubmed/30710055. 30710055 10.1084/jem.20180765 PMC6400537

[17] Han Q, Huang B, Huang Z, Cai J, Gong L, Zhang Y, et al. Tumor cell‑fibroblast heterotypic aggregates in malignant ascites of patients with ovarian cancer. Int J Mol Med. 2019;44:2245–55. http://www.ncbi.nlm.nih.gov/pubmed/31638162. 31638162 10.3892/ijmm.2019.4361 PMC6844628

[18] Wei M, Yang T, Chen X, Wu Y, Deng X, He W, et al. Malignant ascites-derived exosomes promote proliferation and induce carcinoma-associated fibroblasts transition in peritoneal mesothelial cells. Oncotarget. 2017;8:42262–71. http://www.ncbi.nlm.nih.gov/pubmed/28178689. 28178689 10.18632/oncotarget.15040 PMC5522065

[19] Kanamaru R, Ohzawa H, Miyato H, Matsumoto S, Haruta H, Kurashina K, et al. Low density neutrophils (LDN) in postoperative abdominal cavity assist the peritoneal recurrence through the production of neutrophil extracellular traps (NETs). Sci Rep. 2018;8:632 http://www.ncbi.nlm.nih.gov/pubmed/29330531. 29330531 10.1038/s41598-017-19091-2 PMC5766579

[20] Al-Haidari AA, Algethami N, Lepsenyi M, Rahman M, Syk I, Thorlacius H. Neutrophil extracellular traps promote peritoneal metastasis of colon cancer cells. Oncotarget. 2019;10:1238–49. http://www.ncbi.nlm.nih.gov/pubmed/30815227. 30815227 10.18632/oncotarget.26664 PMC6383817

[21] Koppe MJ, Nagtegaal ID, de Wilt JHW, Ceelen WP. Recent insights into the pathophysiology of omental metastases. J Surg Oncol. 2014;110:670–5. http://www.ncbi.nlm.nih.gov/pubmed/24962271. 24962271 10.1002/jso.23681

[22] Sorensen EW, Gerber SA, Sedlacek AL, Rybalko VY, Chan WM, Lord EM. Omental immune aggregates and tumor metastasis within the peritoneal cavity. Immunol Res. 2009;45:185–94. http://www.ncbi.nlm.nih.gov/pubmed/19253004. 19253004 10.1007/s12026-009-8100-2 PMC2891204

[23] Ween MP, Oehler MK, Ricciardelli C. Role of versican, hyaluronan and CD44 in ovarian cancer metastasis. Int J Mol Sci. 2011;12:1009–29. http://www.ncbi.nlm.nih.gov/pubmed/21541039. 21541039 10.3390/ijms12021009 PMC3083686

[24] Nakamura K, Sawada K, Kinose Y, Yoshimura A, Toda A, Nakatsuka E, et al. Exosomes promote ovarian cancer cell invasion through transfer of CD44 to peritoneal mesothelial cells. Mol Cancer Res. 2017;15:78–92. http://www.ncbi.nlm.nih.gov/pubmed/27758876. 27758876 10.1158/1541-7786.MCR-16-0191

[25] Zhang R, Qi F, Shao S, Li G, Feng Y. Human colorectal cancer-derived carcinoma associated fibroblasts promote CD44-mediated adhesion of colorectal cancer cells to endothelial cells by secretion of HGF. Cancer Cell Int. 2019;19:192 http://www.ncbi.nlm.nih.gov/pubmed/31367190. 31367190 10.1186/s12935-019-0914-y PMC6657169

[26] Hilliard TS. The impact of mesothelin in the ovarian cancer tumor microenvironment. Cancers (Basel). 2018;10. http://www.ncbi.nlm.nih.gov/pubmed/30134520. 10.3390/cancers10090277 PMC6162689 30134520

[27] Baldo P, Cecco S. Amatuximab and novel agents targeting mesothelin for solid tumors. Onco Targets Ther. 2017;10:5337–53. https://www.dovepress.com/amatuximab-and-novel-agents-targeting-mesothelin-for-solid-tumors-peer-reviewed-article-OTT. 29184420 10.2147/OTT.S145105 PMC5687483

[28] Lee EK, Liu JF. Antibody-drug conjugates in gynecologic malignancies. Gynecol Oncol. 2019;153:694–702. http://www.ncbi.nlm.nih.gov/pubmed/30929824. 30929824 10.1016/j.ygyno.2019.03.245

[29] Torres K, Pietrzyk Ł, Plewa Z, Załuska-Patel K, Majewski M, Radzikowska E, et al. TGF-β and inflammatory blood markers in prediction of intraperitoneal adhesions. Adv Med Sci. 2018;63:220–3. http://www.ncbi.nlm.nih.gov/pubmed/29223125. 29223125 10.1016/j.advms.2017.11.006

[30] Selgas R, Bajo A, Jiménez-Heffernan JA, Sánchez-Tomero JA, Del Peso G, Aguilera A, et al. Epithelial-to-mesenchymal transition of the mesothelial cell–its role in the response of the peritoneum to dialysis. Nephrol Dial Transpl. 2006;21:ii2–7. http://www.ncbi.nlm.nih.gov/pubmed/16825254. 10.1093/ndt/gfl183 16825254

[31] Dvorak HF. Tumors: wounds that do not heal. Similarities between tumor stroma generation and wound healing. N Engl J Med. 1986;315:1650–9. 10.1056/NEJM198612253152606. 10.1056/NEJM198612253152606 3537791

[32] Ramos C, Oehler R. Clearance of apoptotic cells by neutrophils in inflammation and cancer. Cell Death Discovery. 2024;10:26. 10.1038/s41420-024-01809-7. 10.1038/s41420-024-01809-7 PMC10787834 38218739

[33] Plikus MV, Wang X, Sinha S, Forte E, Thompson SM, Herzog EL, et al. Fibroblasts: origins, definitions, and functions in health and disease. Cell. 2021;184:3852–72. http://www.ncbi.nlm.nih.gov/pubmed/34297930. 34297930 10.1016/j.cell.2021.06.024 PMC8566693

[34] Capobianco A, Cottone L, Monno A, Manfredi AA, Rovere‐Querini P, Rovere-Querini P. The peritoneum: healing, immunity, and diseases. J Pathol. 2017;243:137–47. http://onlinelibrary.wiley.com/doi/10.1002/path.4942/full. 28722107 10.1002/path.4942

[35] Sahai E, Astsaturov I, Cukierman E, DeNardo DG, Egeblad M, Evans RM, et al. A framework for advancing our understanding of cancer-associated fibroblasts. Consensus statement. Nat Rev Cancer. 2020;20:174–86. http://www.nature.com/articles/s41568-019-0238-1. 31980749 10.1038/s41568-019-0238-1 PMC7046529

[36] Chen Y, McAndrews KM, Kalluri R. Clinical and therapeutic relevance of cancer-associated fibroblasts. Nat Rev Clin Oncol. 2021;18:792–804. https://www.nature.com/articles/s41571-021-00546-5. 34489603 10.1038/s41571-021-00546-5 PMC8791784

[37] Simon T, Salhia B. Cancer-associated fibroblast subpopulations with diverse and dynamic roles in the tumor microenvironment. Mol Cancer Res. 2022;20:183–92. http://www.ncbi.nlm.nih.gov/pubmed/34670861. 34670861 10.1158/1541-7786.MCR-21-0282 PMC9306405

[38] Loktev A, Lindner T, Mier W, Debus J, Altmann A, Jäger D, et al. A tumor-imaging method targeting cancer-associated fibroblasts. J Nucl Med. 2018;59:1423–9. http://www.ncbi.nlm.nih.gov/pubmed/29626120. 29626120 10.2967/jnumed.118.210435 PMC6126438

[39] Nurmik M, Ullmann P, Rodriguez F, Haan S, Letellier E. In search of definitions: cancer‐associated fibroblasts and their markers. Int J Cancer. 2020;146:895–905. http://www.ncbi.nlm.nih.gov/pubmed/30734283. 30734283 10.1002/ijc.32193 PMC6972582

[40] Lavie D, Ben-Shmuel A, Erez N, Scherz-Shouval R. Cancer-associated fibroblasts in the single-cell era. Nat Cancer. 2022;3:793–807. http://www.ncbi.nlm.nih.gov/pubmed/35883004. 35883004 10.1038/s43018-022-00411-z PMC7613625

[41] Ma C, Yang C, Peng A, Sun T, Ji X, Mi J, et al. Pan-cancer spatially resolved single-cell analysis reveals the crosstalk between cancer-associated fibroblasts and tumor microenvironment. Mol Cancer. 2023;22:170. http://www.ncbi.nlm.nih.gov/pubmed/37833788. 37833788 10.1186/s12943-023-01876-x PMC10571470

[42] Garrison AT, Bignold RE, Wu X, Johnson JR. Pericytes: the lung-forgotten cell type. Front Physiol. 2023;14:1150028 http://www.ncbi.nlm.nih.gov/pubmed/37035669. 37035669 10.3389/fphys.2023.1150028 PMC10076600

[43] Qian J, Olbrecht S, Boeckx B, Vos H, Laoui D, Etlioglu E, et al. A pan-cancer blueprint of the heterogeneous tumor microenvironment revealed by single-cell profiling. Cell Res. 2020;30:745–62. http://www.nature.com/articles/s41422-020-0355-0. 32561858 10.1038/s41422-020-0355-0 PMC7608385

[44] Shih AJ, Menzin A, Whyte J, Lovecchio J, Liew A, Khalili H, et al. Identification of grade and origin specific cell populations in serous epithelial ovarian cancer by single cell RNA-seq. Orsulic S, editor. PLoS One. 2018;13:e0206785 https://dx.plos.org/10.1371/journal.pone.0206785. 30383866 10.1371/journal.pone.0206785 PMC6211742

[45] Izar B, Tirosh I, Stover EH, Wakiro I, Cuoco MS, Alter I, et al. A single-cell landscape of high-grade serous ovarian cancer. Nat Med. 2020;26:1271–9. https://www.nature.com/articles/s41591-020-0926-0. 32572264 10.1038/s41591-020-0926-0 PMC7723336

[46] Carvalho RF, do Canto LM, Abildgaard C, Aagaard MM, Tronhjem MS, Waldstrøm M, et al. Single-cell and bulk RNA sequencing reveal ligands and receptors associated with worse overall survival in serous ovarian cancer. Cell Commun Signal. 2022;20:176. https://biosignaling.biomedcentral.com/articles/10.1186/s12964-022-00991-4. 36352420 10.1186/s12964-022-00991-4 PMC9648056

[47] Loret N, Vandamme N, De Coninck J, Taminau J, De Clercq K, Blancke G, et al. Distinct transcriptional programs in ascitic and solid cancer cells induce different responses to chemotherapy in high-grade serous ovarian cancer. Mol Cancer Res. 2022;20:1532–47. http://www.ncbi.nlm.nih.gov/pubmed/35749080. 35749080 10.1158/1541-7786.MCR-21-0565

[48] Coward J, Kulbe H, Chakravarty P, Leader D, Vassileva V, Leinster DA, et al. Interleukin-6 as a therapeutic target in human ovarian cancer. Clin Cancer Res. 2011;17:6083–96. https://aacrjournals.org/clincancerres/article/17/18/6083/76404/Interleukin-6-as-a-Therapeutic-Target-in-Human. 21795409 10.1158/1078-0432.CCR-11-0945 PMC3182554

[49] Lim H, Kim SI, Kim EN, Lee M, Lee C, Kim JW, et al. Tissue expression and prognostic role of CXCL12 and CXCR4 in high-grade serous ovarian carcinoma. Anticancer Res. 2023;43:3331–40. http://www.ncbi.nlm.nih.gov/pubmed/37351997. 37351997 10.21873/anticanres.16509

[50] Zhang K, Erkan EP, Jamalzadeh S, Dai J, Andersson N, Kaipio K, et al. Longitudinal single-cell RNA-seq analysis reveals stress-promoted chemoresistance in metastatic ovarian cancer. Sci Adv. 2022;8:eabm1831. http://www.ncbi.nlm.nih.gov/pubmed/35196078. 10.1126/sciadv.abm1831 PMC8865800 35196078

[51] Olbrecht S, Busschaert P, Qian J, Vanderstichele A, Loverix L, Van Gorp T, et al. High-grade serous tubo-ovarian cancer refined with single-cell RNA sequencing: specific cell subtypes influence survival and determine molecular subtype classification. Genome Med. 2021;13:111. https://genomemedicine.biomedcentral.com/articles/10.1186/s13073-021-00922-x. 34238352 10.1186/s13073-021-00922-x PMC8268616

[52] Deng Y, Tan Y, Zhou D, Bai Y, Cao T, Zhong C, et al. Single-cell RNA-sequencing atlas reveals the tumor microenvironment of metastatic high-grade serous ovarian carcinoma. Front Immunol. 2022;13. https://www.frontiersin.org/articles/10.3389/fimmu.2022.923194/full. 10.3389/fimmu.2022.923194 PMC9354882 35935940

[53] Kan T, Zhang S, Zhou S, Zhang Y, Zhao Y, Gao Y, et al. Single-cell RNA-seq recognized the initiator of epithelial ovarian cancer recurrence. Oncogene. 2022;41:895–906. https://www.nature.com/articles/s41388-021-02139-z. 34992217 10.1038/s41388-021-02139-z

[54] Cords L, Tietscher S, Anzeneder T, Langwieder C, Rees M, de Souza N, et al. Cancer-associated fibroblast classification in single-cell and spatial proteomics data. Nat Commun. 2023;14:4294. https://www.nature.com/articles/s41467-023-39762-1. 37463917 10.1038/s41467-023-39762-1 PMC10354071

[55] Xu J, Fang Y, Chen K, Li S, Tang S, Ren Y, et al. Single-cell RNA sequencing reveals the tissue architecture in human high-grade serous ovarian cancer. Clin Cancer Res. 2022;28:3590–602. https://aacrjournals.org/clincancerres/article/28/16/3590/707396/Single-Cell-RNA-Sequencing-Reveals-the-Tissue. 35675036 10.1158/1078-0432.CCR-22-0296 PMC9662915

[56] Jiang H, Yu D, Yang P, Guo R, Kong M, Gao Y, et al. Revealing the transcriptional heterogeneity of organ‐specific metastasis in human gastric cancer using single‐cell RNA Sequencing. Clin Transl Med. 2022;12. https://onlinelibrary.wiley.com/doi/10.1002/ctm2.730. 10.1002/ctm2.730 PMC8858624 35184420

[57] Wang R, Song S, Qin J, Yoshimura K, Peng F, Chu Y, et al. Evolution of immune and stromal cell states and ecotypes during gastric adenocarcinoma progression. Cancer Cell. 2023;41:1407–26.e9. https://linkinghub.elsevier.com/retrieve/pii/S1535610823002155. 37419119 10.1016/j.ccell.2023.06.005 PMC10528152

[58] Arina A, Idel C, Hyjek EM, Alegre ML, Wang Y, Bindokas VP, et al. Tumor-associated fibroblasts predominantly come from local and not circulating precursors. Proc Natl Acad Sci USA. 2016;113:7551–6. http://www.ncbi.nlm.nih.gov/pubmed/27317748. 27317748 10.1073/pnas.1600363113 PMC4941507

[59] Yáñez-Mó M, Lara-Pezzi E, Selgas R, Ramírez-Huesca M, Domínguez-Jiménez C, Jiménez-Heffernan JA, et al. Peritoneal dialysis and epithelial-to-mesenchymal transition of mesothelial cells. N Engl J Med. 2003;348:403–13. http://www.ncbi.nlm.nih.gov/pubmed/12556543. 12556543 10.1056/NEJMoa020809

[60] Rynne-Vidal A, Jiménez-Heffernan JA, Fernández-Chacón C, López-Cabrera M, Sandoval P. The mesothelial origin of carcinoma associated-fibroblasts in peritoneal metastasis. Cancers (Basel). 2015;7:1994–2011. http://www.ncbi.nlm.nih.gov/pubmed/26426054. 26426054 10.3390/cancers7040872 PMC4695872

[61] Hadis U, Wahl B, Schulz O, Hardtke-Wolenski M, Schippers A, Wagner N, et al. Intestinal tolerance requires gut homing and expansion of FoxP3+ regulatory T cells in the lamina propria. Immunity. 2011;34:237–46. 21333554 10.1016/j.immuni.2011.01.016

[62] Sandoval P, Jiménez-Heffernan JA, Rynne-Vidal Á, Pérez-Lozano ML, Gilsanz Á, Ruiz-Carpio V, et al. Carcinoma-associated fibroblasts derive from mesothelial cells via mesothelial-to-mesenchymal transition in peritoneal metastasis. J Pathol. 2013;231:517–31. http://www.ncbi.nlm.nih.gov/pubmed/24114721. 24114721 10.1002/path.4281

[63] Lv ZD, Na D, Ma XY, Zhao C, Zhao WJ, Xu HM. Human peritoneal mesothelial cell transformation into myofibroblasts in response to TGF-ß1 in vitro. Int J Mol Med. 2011;27:187–93. http://www.ncbi.nlm.nih.gov/pubmed/21152863. 21152863 10.3892/ijmm.2010.574

[64] Satoyoshi R, Aiba N, Yanagihara K, Yashiro M, Tanaka M. Tks5 activation in mesothelial cells creates invasion front of peritoneal carcinomatosis. Oncogene. 2015;34:3176–87. http://www.ncbi.nlm.nih.gov/pubmed/25088196. 25088196 10.1038/onc.2014.246

[65] Miao ZF, Zhao TT, Wang ZN, Miao F, Xu YY, Mao XY, et al. Tumor-associated mesothelial cells are negative prognostic factors in gastric cancer and promote peritoneal dissemination of adherent gastric cancer cells by chemotaxis. Tumour Biol. 2014;35:6105–11. http://www.ncbi.nlm.nih.gov/pubmed/24615523. 24615523 10.1007/s13277-014-1808-1

[66] Gordillo CH, Sandoval P, Muñoz-Hernández P, Pascual-Antón L, López-Cabrera M, Jiménez-Heffernan JA. Mesothelial-to-mesenchymal transition contributes to the generation of carcinoma-associated fibroblasts in locally advanced primary colorectal carcinomas. Cancers (Basel). 2020;12:499 https://www.mdpi.com/2072-6694/12/2/499. 32098058 10.3390/cancers12020499 PMC7072259

[67] Rynne-Vidal A, Au-Yeung CL, Jiménez-Heffernan JA, Pérez-Lozano ML, Cremades-Jimeno L, Bárcena C, et al. Mesothelial-to-mesenchymal transition as a possible therapeutic target in peritoneal metastasis of ovarian cancer. J Pathol. 2017;242:140–51. http://www.ncbi.nlm.nih.gov/pubmed/28247413. 28247413 10.1002/path.4889 PMC5468005

[68] Mikuła-Pietrasik J, Sosińska P, Kucińska M, Murias M, Maksin K, Malińska A, et al. Peritoneal mesothelium promotes the progression of ovarian cancer cells in vitro and in a mice xenograft model in vivo. Cancer Lett. 2014;355:310–5. http://www.ncbi.nlm.nih.gov/pubmed/25301450. 25301450 10.1016/j.canlet.2014.09.041

[69] Tsukada T, Fushida S, Harada S, Yagi Y, Kinoshita J, Oyama K, et al. The role of human peritoneal mesothelial cells in the fibrosis and progression of gastric cancer. Int J Oncol. 2012;41:476–82. http://www.ncbi.nlm.nih.gov/pubmed/22614335. 22614335 10.3892/ijo.2012.1490 PMC3582882

[70] Demuytere J, Ceelen W, Van Dorpe J, Hoorens A. The role of the peritoneal microenvironment in the pathogenesis of colorectal peritoneal carcinomatosis. Exp Mol Pathol. 2020;115:104442 http://www.ncbi.nlm.nih.gov/pubmed/32305340. 32305340 10.1016/j.yexmp.2020.104442

[71] Huang H, Wang Z, Zhang Y, Pradhan RN, Ganguly D, Chandra R, et al. Mesothelial cell-derived antigen-presenting cancer-associated fibroblasts induce expansion of regulatory T cells in pancreatic cancer. Cancer Cell. 2022;40:656–73.e7. http://www.ncbi.nlm.nih.gov/pubmed/5523176. 35523176 10.1016/j.ccell.2022.04.011 PMC9197998

[72] Jiménez-Heffernan JA, Aguilera A, Aroeira LS, Lara-Pezzi E, Bajo MA, del Peso G. et al. Immunohistochemical characterization of fibroblast subpopulations in normal peritoneal tissue and in peritoneal dialysis-induced fibrosis. Virchows Arch. 2004;444:247–56. http://www.ncbi.nlm.nih.gov/pubmed/14749928. 14749928 10.1007/s00428-003-0963-3

[73] Becker LM, O’Connell JT, Vo AP, Cain MP, Tampe D, Bizarro L, et al. Epigenetic reprogramming of cancer-associated fibroblasts deregulates glucose metabolism and facilitates progression of breast cancer. Cell Rep. 2020;31:107701 https://linkinghub.elsevier.com/retrieve/pii/S2211124720306719. 32492417 10.1016/j.celrep.2020.107701 PMC7339325

[74] Bhagat TD, Von Ahrens D, Dawlaty M, Zou Y, Baddour J, Achreja A, et al. Lactate-mediated epigenetic reprogramming regulates formation of human pancreatic cancer-associated fibroblasts. Elife. 2019;8. http://www.ncbi.nlm.nih.gov/pubmed/31663852. 10.7554/eLife.50663 PMC6874475 31663852

[75] Chen YT, Chang YT, Pan SY, Chou YH, Chang FC, Yeh PY, et al. Lineage tracing reveals distinctive fates for mesothelial cells and submesothelial fibroblasts during peritoneal injury. J Am Soc Nephrol. 2014;25:2847–58. http://www.ncbi.nlm.nih.gov/pubmed/24854266. 24854266 10.1681/ASN.2013101079 PMC4243351

[76] Kobayashi H, Gieniec KA, Lannagan TRM, Wang T, Asai N, Mizutani Y, et al. The origin and contribution of cancer-associated fibroblasts in colorectal carcinogenesis. Gastroenterol. 2022;162:890–906. http://www.ncbi.nlm.nih.gov/pubmed/34883119. 10.1053/j.gastro.2021.11.037 PMC8881386 34883119

[77] Calon A, Espinet E, Palomo-Ponce S, Tauriello DVFVF, Iglesias M, Céspedes MVV, et al. Dependency of colorectal cancer on a TGF-β-driven program in stromal cells for metastasis initiation. Cancer Cell. 2012;22:571–84. http://www.ncbi.nlm.nih.gov/pubmed/23153532. 23153532 10.1016/j.ccr.2012.08.013 PMC3512565

[78] Calvo F, Ege N, Grande-Garcia A, Hooper S, Jenkins RP, Chaudhry SI, et al. Mechanotransduction and YAP-dependent matrix remodelling is required for the generation and maintenance of cancer-associated fibroblasts. Nat Cell Biol. 2013;15:637–46. http://www.ncbi.nlm.nih.gov/pubmed/23708000. 23708000 10.1038/ncb2756 PMC3836234

[79] Sung SY, Hsieh CL, Law A, Zhau HE, Pathak S, Multani AS, et al. Coevolution of prostate cancer and bone stroma in three-dimensional coculture: implications for cancer growth and metastasis. Cancer Res. 2008;68:9996–10003. http://www.ncbi.nlm.nih.gov/pubmed/19047182. 19047182 10.1158/0008-5472.CAN-08-2492 PMC3105756

[80] Cai J, Tang H, Xu L, Wang X, Yang C, Ruan S, et al. Fibroblasts in omentum activated by tumor cells promote ovarian cancer growth, adhesion and invasiveness. Carcinogenesis. 2012;33:20–9. http://www.ncbi.nlm.nih.gov/pubmed/22021907. 22021907 10.1093/carcin/bgr230

[81] Han Q, Tan S, Gong L, Li G, Wu Q, Chen L, et al. Omental cancer-associated fibroblast-derived exosomes with low microRNA-29c-3p promote ovarian cancer peritoneal metastasis. Cancer Sci. 2023;114:1929–42. http://www.ncbi.nlm.nih.gov/pubmed/36644823. 36644823 10.1111/cas.15726 PMC10154903

[82] Hosaka K, Yang Y, Seki T, Fischer C, Dubey O, Fredlund E, et al. Pericyte-fibroblast transition promotes tumor growth and metastasis. Proc Natl Acad Sci USA. 2016;113:E5618–27. http://www.ncbi.nlm.nih.gov/pubmed/27608497. 27608497 10.1073/pnas.1608384113 PMC5035870

[83] Ning X, Zhang H, Wang C, Song X. Exosomes released by gastric cancer cells induce transition of pericytes into cancer-associated fibroblasts. Med Sci Monit. 2018;24:2350–9. http://www.ncbi.nlm.nih.gov/pubmed/29668670. 29668670 10.12659/MSM.906641 PMC5922989

[84] Shao Q, Sun C, Zhang Q, Liu J, Xia Y, Jin B, et al. Macrophages regulates the transition of pericyte to peritoneal fibrosis through the GSDMD/IL-1β axis. Int Immunopharmacol. 2021;101:108323 http://www.ncbi.nlm.nih.gov/pubmed/34749292. 34749292 10.1016/j.intimp.2021.108323

[85] Kuppe C, Ibrahim MM, Kranz J, Zhang X, Ziegler S, Perales-Patón J, et al. Decoding myofibroblast origins in human kidney fibrosis. Nature. 2021;589:281–6. http://www.ncbi.nlm.nih.gov/pubmed/33176333. 33176333 10.1038/s41586-020-2941-1 PMC7611626

[86] Humphreys BD, Lin SL, Kobayashi A, Hudson TE, Nowlin BT, Bonventre JV, et al. Fate tracing reveals the pericyte and not epithelial origin of myofibroblasts in kidney fibrosis. Am J Pathol. 2010;176:85–97. http://www.ncbi.nlm.nih.gov/pubmed/20008127. 20008127 10.2353/ajpath.2010.090517 PMC2797872

[87] Zeisberg EM, Potenta S, Xie L, Zeisberg M, Kalluri R. Discovery of endothelial to mesenchymal transition as a source for carcinoma-associated fibroblasts. Cancer Res. 2007;67:10123–8. http://www.ncbi.nlm.nih.gov/pubmed/17974953. 17974953 10.1158/0008-5472.CAN-07-3127

[88] Wawro ME, Chojnacka K, Wieczorek-Szukała K, Sobierajska K, Niewiarowska J. Invasive colon cancer cells induce transdifferentiation of endothelium to cancer-associated fibroblasts through microtubules enriched in Tubulin-β3. Int J Mol Sci. 2018;20. http://www.ncbi.nlm.nih.gov/pubmed/30583584. 10.3390/ijms20010053 PMC6337286 30583584

[89] Zeisberg EM, Potenta SE, Sugimoto H, Zeisberg M, Kalluri R. Fibroblasts in kidney fibrosis emerge via endothelial-to-mesenchymal transition. J Am Soc Nephrol. 2008;19:2282–7. http://www.ncbi.nlm.nih.gov/pubmed/18987304. 18987304 10.1681/ASN.2008050513 PMC2588112

[90] Strippoli R, Loureiro J, Moreno V, Benedicto I, Pérez Lozano ML, Barreiro O, et al. Caveolin-1 deficiency induces a MEK-ERK1/2-Snail-1-dependent epithelial-mesenchymal transition and fibrosis during peritoneal dialysis. EMBO Mol Med. 2015;7:102–23. http://www.ncbi.nlm.nih.gov/pubmed/25550395. 25550395 10.15252/emmm.201404127 PMC4309670

[91] Nieman KM, Romero IL, Van Houten B, Lengyel E. Adipose tissue and adipocytes support tumorigenesis and metastasis. Biochim Biophys Acta. 2013;1831:1533–41. http://www.ncbi.nlm.nih.gov/pubmed/23500888. 23500888 10.1016/j.bbalip.2013.02.010 PMC3742583

[92] Natsume M, Shimura T, Iwasaki H, Okuda Y, Hayashi K, Takahashi S, et al. Omental adipocytes promote peritoneal metastasis of gastric cancer through the CXCL2-VEGFA axis. Br J Cancer. 2020;123:459–70. http://www.ncbi.nlm.nih.gov/pubmed/32439934. 32439934 10.1038/s41416-020-0898-3 PMC7403422

[93] Hamabe-Horiike T, Harada SI, Yoshida K, Kinoshita J, Yamaguchi T, Fushida S. Adipocytes contribute to tumor progression and invasion of peritoneal metastasis by interacting with gastric cancer cells as cancer associated fibroblasts. Cancer Rep (Hoboken NJ). 2023;6:e1647 http://www.ncbi.nlm.nih.gov/pubmed/35691615. 10.1002/cnr2.1647 PMC9875653 35691615

[94] Chandler EM, Seo BR, Califano JP, Andresen Eguiluz RC, Lee JS, Yoon CJ, et al. Implanted adipose progenitor cells as physicochemical regulators of breast cancer. Proc Natl Acad Sci USA. 2012;109:9786–91. http://www.ncbi.nlm.nih.gov/pubmed/22665775. 22665775 10.1073/pnas.1121160109 PMC3382522

[95] Toyoda M, Matsubara Y, Lin K, Sugimachi K, Furue M. Characterization and comparison of adipose tissue-derived cells from human subcutaneous and omental adipose tissues. Cell Biochem Funct. 2009;27:440–7. http://www.ncbi.nlm.nih.gov/pubmed/19691084. 19691084 10.1002/cbf.1591

[96] Zhang Y, Daquinag A, Traktuev DO, Amaya-Manzanares F, Simmons PJ, March KL, et al. White adipose tissue cells are recruited by experimental tumors and promote cancer progression in mouse models. Cancer Res. 2009;69:5259–66. http://www.ncbi.nlm.nih.gov/pubmed/19491274. 19491274 10.1158/0008-5472.CAN-08-3444 PMC3857703

[97] Miyazaki Y, Oda T, Inagaki Y, Kushige H, Saito Y, Mori N, et al. Adipose-derived mesenchymal stem cells differentiate into heterogeneous cancer-associated fibroblasts in a stroma-rich xenograft model. Sci Rep. 2021;11:4690 http://www.ncbi.nlm.nih.gov/pubmed/33633222. 33633222 10.1038/s41598-021-84058-3 PMC7907195

[98] Kitayama J, Emoto S, Yamaguchi H, Ishigami H, Watanabe T. CD90+ mesothelial-like cells in peritoneal fluid promote peritoneal metastasis by forming a tumor permissive microenvironment. PLoS One. 2014;9:e86516 http://www.ncbi.nlm.nih.gov/pubmed/24466130. 24466130 10.1371/journal.pone.0086516 PMC3897715

[99] Nowicka A, Marini FC, Solley TN, Elizondo PB, Zhang Y, Sharp HJ, et al. Human omental-derived adipose stem cells increase ovarian cancer proliferation, migration, and chemoresistance. PLoS One. 2013;8:e81859 http://www.ncbi.nlm.nih.gov/pubmed/24312594. 24312594 10.1371/journal.pone.0081859 PMC3847080

[100] Chu Y, Tang H, Guo Y, Guo J, Huang B, Fang F, et al. Adipose-derived mesenchymal stem cells promote cell proliferation and invasion of epithelial ovarian cancer. Exp Cell Res. 2015;337:16–27. http://www.ncbi.nlm.nih.gov/pubmed/26209607. 26209607 10.1016/j.yexcr.2015.07.020

[101] Tang H, Chu Y, Huang Z, Cai J, Wang Z. The metastatic phenotype shift toward myofibroblast of adipose-derived mesenchymal stem cells promotes ovarian cancer progression. Carcinogenesis. 2020;41:182–93. http://www.ncbi.nlm.nih.gov/pubmed/31046126. 31046126 10.1093/carcin/bgz083

[102] Jeon ES, Moon HJ, Lee MJ, Song HY, Kim YM, Cho M, et al. Cancer-derived lysophosphatidic acid stimulates differentiation of human mesenchymal stem cells to myofibroblast-like cells. Stem Cells. 2008;26:789–97. http://www.ncbi.nlm.nih.gov/pubmed/18065393. 18065393 10.1634/stemcells.2007-0742

[103] Kidd S, Spaeth E, Watson K, Burks J, Lu H, Klopp A, et al. Origins of the tumor microenvironment: quantitative assessment of adipose-derived and bone marrow-derived stroma. PLoS One. 2012;7:e30563 http://www.ncbi.nlm.nih.gov/pubmed/22363446. 22363446 10.1371/journal.pone.0030563 PMC3282707

[104] Meza-Perez S, Randall TD. Immunological functions of the omentum. Trends Immunol. 2017;38:526–36. https://linkinghub.elsevier.com/retrieve/pii/S1471490617300522. 28579319 10.1016/j.it.2017.03.002 PMC5812451

[105] Ito M, Nakano M, Ariyama H, Yamaguchi K, Tanaka R, Semba Y, et al. Macrophages are primed to transdifferentiate into fibroblasts in malignant ascites and pleural effusions. Cancer Lett. 2022;532:215597 http://www.ncbi.nlm.nih.gov/pubmed/35150810. 35150810 10.1016/j.canlet.2022.215597

[106] Ishigaki K, Kumano K, Fujita K, Ueno H. Cellular basis of omentum activation and expansion revealed by single-cell RNA sequencing using a parabiosis model. Sci Rep. 2021;11:13958 http://www.ncbi.nlm.nih.gov/pubmed/34230565. 34230565 10.1038/s41598-021-93330-5 PMC8260800

[107] Terai S, Fushida S, Tsukada T, Kinoshita J, Oyama K, Okamoto K, et al. Bone marrow derived “fibrocytes” contribute to tumor proliferation and fibrosis in gastric cancer. Gastric Cancer. 2015;18:306–13. http://www.ncbi.nlm.nih.gov/pubmed/24792410. 24792410 10.1007/s10120-014-0380-0 PMC4371822

[108] Bellows CF, Zhang Y, Chen J, Frazier ML, Kolonin MG. Circulation of progenitor cells in obese and lean colorectal cancer patients. Cancer Epidemiol Biomark Prev. 2011;20:2461–8. http://www.ncbi.nlm.nih.gov/pubmed/21930958. 10.1158/1055-9965.EPI-11-0556 PMC5470315 21930958

[109] Direkze NC, Hodivala-Dilke K, Jeffery R, Hunt T, Poulsom R, Oukrif D, et al. Bone marrow contribution to tumor-associated myofibroblasts and fibroblasts. Cancer Res. 2004;64:8492–5. http://www.ncbi.nlm.nih.gov/pubmed/15574751. 15574751 10.1158/0008-5472.CAN-04-1708

[110] Mishra PJ, Mishra PJ, Humeniuk R, Medina DJ, Alexe G, Mesirov JP, et al. Carcinoma-associated fibroblast-like differentiation of human mesenchymal stem cells. Cancer Res. 2008;68:4331–9. http://www.ncbi.nlm.nih.gov/pubmed/18519693. 18519693 10.1158/0008-5472.CAN-08-0943 PMC2725025

[111] Quante M, Tu SP, Tomita H, Gonda T, Wang SSW, Takashi S, et al. Bone marrow-derived myofibroblasts contribute to the mesenchymal stem cell niche and promote tumor growth. Cancer Cell. 2011;19:257–72. http://www.ncbi.nlm.nih.gov/pubmed/21316604. 21316604 10.1016/j.ccr.2011.01.020 PMC3060401

[112] Worthley DL, Ruszkiewicz A, Davies R, Moore S, Nivison-Smith I, Bik L, et al. Human gastrointestinal neoplasia-associated myofibroblasts can develop from bone marrow-derived cells following allogeneic stem cell transplantation. Stem Cells. 2009;27:1463–8. http://www.ncbi.nlm.nih.gov/pubmed/19492298. 19492298 10.1002/stem.63

[113] Radisky DC, Kenny PA, Bissell MJ. Fibrosis and cancer: do myofibroblasts come also from epithelial cells via EMT? J Cell Biochem. 2007;101:830–9. http://www.ncbi.nlm.nih.gov/pubmed/17211838. 17211838 10.1002/jcb.21186 PMC2838476

[114] Nair N, Calle AS, Zahra MH, Prieto-Vila M, Oo AKK, Hurley L, et al. A cancer stem cell model as the point of origin of cancer-associated fibroblasts in tumor microenvironment. Sci Rep. 2017;7:6838 http://www.ncbi.nlm.nih.gov/pubmed/28754894. 28754894 10.1038/s41598-017-07144-5 PMC5533745

[115] Kurose K, Gilley K, Matsumoto S, Watson PH, Zhou XP, Eng C. Frequent somatic mutations in PTEN and TP53 are mutually exclusive in the stroma of breast carcinomas. Nat Genet. 2002;32:355–7. http://www.ncbi.nlm.nih.gov/pubmed/12379854. 12379854 10.1038/ng1013

[116] Yu S, Yang R, Xu T, Li X, Wu S, Zhang J. Cancer-associated fibroblasts-derived FMO2 as a biomarker of macrophage infiltration and prognosis in epithelial ovarian cancer. Gynecol Oncol. 2022;167:342–53. https://linkinghub.elsevier.com/retrieve/pii/S0090825822005893. 36114029 10.1016/j.ygyno.2022.09.003

[117] Fujikake K, Kajiyama H, Yoshihara M, Nishino K, Yoshikawa N, Utsumi F, et al. A novel mechanism of neovascularization in peritoneal dissemination via cancer-associated mesothelial cells affected by TGF-β derived from ovarian cancer. Oncol Rep. 2018;39:193–200. http://www.ncbi.nlm.nih.gov/pubmed/29192324. 29192324 10.3892/or.2017.6104

[118] Mo Y, Leung LL, Mak CSL, Wang X, Chan WS, Hui LMN, et al. Tumor-secreted exosomal miR-141 activates tumor-stroma interactions and controls premetastatic niche formation in ovarian cancer metastasis. Mol Cancer. 2023;22:4 http://www.ncbi.nlm.nih.gov/pubmed/36624516. 36624516 10.1186/s12943-022-01703-9 PMC9827705

[119] Iyoshi S, Yoshihara M, Nakamura K, Sugiyama M, Koya Y, Kitami K, et al. Pro-tumoral behavior of omental adipocyte-derived fibroblasts in tumor microenvironment at the metastatic site of ovarian cancer. Int J Cancer. 2021;149:1961–72. http://www.ncbi.nlm.nih.gov/pubmed/34469585. 34469585 10.1002/ijc.33770

[120] Cho JA, Park H, Lim EH, Lee KW. Exosomes from breast cancer cells can convert adipose tissue-derived mesenchymal stem cells into myofibroblast-like cells. Int J Oncol. 2012;40:130–8. http://www.ncbi.nlm.nih.gov/pubmed/21904773. 21904773 10.3892/ijo.2011.1193

[121] Cho JA, Park H, Lim EH, Kim KH, Choi JS, Lee JH, et al. Exosomes from ovarian cancer cells induce adipose tissue-derived mesenchymal stem cells to acquire the physical and functional characteristics of tumor-supporting myofibroblasts. Gynecol Oncol. 2011;123:379–86. http://www.ncbi.nlm.nih.gov/pubmed/21903249. 21903249 10.1016/j.ygyno.2011.08.005

[122] Saito H, Fushida S, Harada S, Miyashita T, Oyama K, Yamaguchi T, et al. Importance of human peritoneal mesothelial cells in the progression, fibrosis, and control of gastric cancer: inhibition of growth and fibrosis by tranilast. Gastric Cancer. 2018;21:55–67. http://www.ncbi.nlm.nih.gov/pubmed/28540637. 28540637 10.1007/s10120-017-0726-5 PMC5741788

[123] Zhu Q, Zhang X, Zhang L, Li W, Wu H, Yuan X, et al. The IL-6-STAT3 axis mediates a reciprocal crosstalk between cancer-derived mesenchymal stem cells and neutrophils to synergistically prompt gastric cancer progression. Cell Death Dis. 2014;5:e1295. http://www.ncbi.nlm.nih.gov/pubmed/24946088. 24946088 10.1038/cddis.2014.263 PMC4611735

[124] Gu J, Qian H, Shen L, Zhang X, Zhu W, Huang L, et al. Gastric cancer exosomes trigger differentiation of umbilical cord derived mesenchymal stem cells to carcinoma-associated fibroblasts through TGF-β/Smad pathway. PLoS One. 2012;7:e52465 http://www.ncbi.nlm.nih.gov/pubmed/24946088. 23285052 10.1371/journal.pone.0052465 PMC3527492

[125] Kojima M, Higuchi Y, Yokota M, Ishii G, Saito N, Aoyagi K, et al. Human subperitoneal fibroblast and cancer cell interaction creates microenvironment that enhances tumor progression and metastasis. PLoS One. 2014;9:e88018 http://www.ncbi.nlm.nih.gov/pubmed/24505356. 24505356 10.1371/journal.pone.0088018 PMC3913740

[126] Peng Y, Li Z, Li Z. GRP78 secreted by tumor cells stimulates differentiation of bone marrow mesenchymal stem cells to cancer-associated fibroblasts. Biochem Biophys Res Commun. 2013;440:558–63. http://www.ncbi.nlm.nih.gov/pubmed/24113381. 24113381 10.1016/j.bbrc.2013.09.108

[127] Peng Y, Li Z, Yang P, Newton IP, Ren H, Zhang L, et al. Direct contacts with colon cancer cells regulate the differentiation of bone marrow mesenchymal stem cells into tumor associated fibroblasts. Biochem Biophys Res Commun. 2014;451:68–73. http://www.ncbi.nlm.nih.gov/pubmed/25063031. 25063031 10.1016/j.bbrc.2014.07.074

[128] Klopp AH, Zhang Y, Solley T, Amaya-Manzanares F, Marini F, Andreeff M, et al. Omental adipose tissue-derived stromal cells promote vascularization and growth of endometrial tumors. Clin Cancer Res. 2012;18:771–82. http://www.ncbi.nlm.nih.gov/pubmed/22167410. 22167410 10.1158/1078-0432.CCR-11-1916 PMC3481843

[129] Li M, Li X, Zhao L, Zhou J, Cheng Y, Xu B, et al. Spontaneous formation of tumorigenic hybrids between human omental adipose-derived stromal cells and endometrial cancer cells increased motility and heterogeneity of cancer cells. Cell Cycle. 2019;18:320–32. http://www.ncbi.nlm.nih.gov/pubmed/30636489. 30636489 10.1080/15384101.2019.1568743 PMC6380430

[130] Zoico E, Darra E, Rizzatti V, Budui S, Franceschetti G, Mazzali G, et al. Adipocytes WNT5a mediated dedifferentiation: a possible target in pancreatic cancer microenvironment. Oncotarget. 2016;7:20223–35. http://www.ncbi.nlm.nih.gov/pubmed/26958939. 26958939 10.18632/oncotarget.7936 PMC4991449

[131] Namvar S, Woolf AS, Zeef LA, Wilm T, Wilm B, Herrick SE. Functional molecules in mesothelial-to-mesenchymal transition revealed by transcriptome analyses. J Pathol. 2018;245:491–501. http://www.ncbi.nlm.nih.gov/pubmed/29774544. 29774544 10.1002/path.5101 PMC6055603

[132] Si M, Wang Q, Li Y, Lin H, Luo D, Zhao W, et al. Inhibition of hyperglycolysis in mesothelial cells prevents peritoneal fibrosis. Sci Transl Med. 2019;11. http://www.ncbi.nlm.nih.gov/pubmed/31167927. 10.1126/scitranslmed.aav5341 31167927

[133] Loureiro J, Schilte M, Aguilera A, Albar-Vizcaíno P, Ramírez-Huesca M, Pérez-Lozano ML, et al. BMP-7 blocks mesenchymal conversion of mesothelial cells and prevents peritoneal damage induced by dialysis fluid exposure. Nephrol Dial Transpl. 2010;25:1098–108. http://www.ncbi.nlm.nih.gov/pubmed/20067910. 10.1093/ndt/gfp618 20067910

[134] Yang AH, Chen JY, Lin JK. Myofibroblastic conversion of mesothelial cells. Kidney Int. 2003;63:1530–9. http://www.ncbi.nlm.nih.gov/pubmed/12631370. 12631370 10.1046/j.1523-1755.2003.00861.x

[135] Shang J, He Q, Chen Y, Yu D, Sun L, Cheng G, et al. miR-15a-5p suppresses inflammation and fibrosis of peritoneal mesothelial cells induced by peritoneal dialysis via targeting VEGFA. J Cell Physiol. 2019;234:9746–55. http://www.ncbi.nlm.nih.gov/pubmed/30362573. 30362573 10.1002/jcp.27660

[136] Tian M, Zhang L, Wang Y, Deng M, Peng C, Liang W, et al. Loss of JNK-associated leucine zipper protein promotes peritoneal dialysis-related peritoneal fibrosis. Kidney Dis (Basel Switz). 2022;8:168–79. http://www.ncbi.nlm.nih.gov/pubmed/35527988. 10.1159/000521564 PMC9021628 35527988

[137] Del Peso G, Jiménez-Heffernan JA, Bajo MA, Aroeira LS, Aguilera A, Fernández-Perpén A, et al. Epithelial-to-mesenchymal transition of mesothelial cells is an early event during peritoneal dialysis and is associated with high peritoneal transport. Kidney Int Suppl. 2008;S26–33. http://www.ncbi.nlm.nih.gov/pubmed/18379544. 10.1038/sj.ki.5002598 18379544

[138] Aroeira LS, Aguilera A, Selgas R, Ramírez-Huesca M, Pérez-Lozano ML, Cirugeda A, et al. Mesenchymal conversion of mesothelial cells as a mechanism responsible for high solute transport rate in peritoneal dialysis: role of vascular endothelial growth factor. Am J Kidney Dis. 2005;46:938–48. http://www.ncbi.nlm.nih.gov/pubmed/16253736. 16253736 10.1053/j.ajkd.2005.08.011

[139] Strippoli R, Benedicto I, Pérez Lozano ML, Cerezo A, López-Cabrera M, del Pozo MA. Epithelial-to-mesenchymal transition of peritoneal mesothelial cells is regulated by an ERK/NF-kappaB/Snail1 pathway. Dis Model Mech. 2008;1:264–74. http://www.ncbi.nlm.nih.gov/pubmed/19093035. 19093035 10.1242/dmm.001321 PMC2590814

[140] Loureiro J, Aguilera A, Selgas R, Sandoval P, Albar-Vizcaíno P, Pérez-Lozano ML, et al. Blocking TGF-β1 protects the peritoneal membrane from dialysate-induced damage. J Am Soc Nephrol. 2011;22:1682–95. http://www.ncbi.nlm.nih.gov/pubmed/21742730. 21742730 10.1681/ASN.2010111197 PMC3171939

[141] Okada H, Inoue T, Kanno Y, Kobayashi T, Watanabe Y, Ban S, et al. Selective depletion of fibroblasts preserves morphology and the functional integrity of peritoneum in transgenic mice with peritoneal fibrosing syndrome. Kidney Int. 2003;64:1722–32. http://www.ncbi.nlm.nih.gov/pubmed/14531805. 14531805 10.1046/j.1523-1755.2003.00290.x

[142] Mooney JE, Rolfe BE, Osborne GW, Sester DP, van Rooijen N, Campbell GR, et al. Cellular plasticity of inflammatory myeloid cells in the peritoneal foreign body response. Am J Pathol. 2010;176:369–80. http://www.ncbi.nlm.nih.gov/pubmed/20008135. 20008135 10.2353/ajpath.2010.090545 PMC2797897

[143] Ninomiya K, Takahashi A, Fujioka Y, Ishikawa Y, Yokoyama M. Transforming growth factor-beta signaling enhances transdifferentiation of macrophages into smooth muscle-like cells. Hypertens Res. 2006;29:269–76. http://www.ncbi.nlm.nih.gov/pubmed/16778334. 16778334 10.1291/hypres.29.269

