# Supplementary material for: Metastasis-associated fibroblasts in peritoneal surface malignancies
Source: Br J Cancer. 2024 May 23;131(3):407–19. doi: 10.1038/s41416-024-02717-4 (PMC11300623; doi:10.1038/s41416-024-02717-4)
Supplement: Supplementary file 1 — Supplementary Table 1 [file 41416_2024_2717_MOESM1_ESM.pdf]

Primary disease	Tumor stages	Sample Type	Fibroblast functional types	Differentially expressed markers	Reference
**PSM-Ovarian cancer**					
HGSOC (n=3)<br>LGSOC (n=1)	IIIA (n=1) , IIIB (n=1), IIIC (n=2)	Solid tumor	iCAF (metastatic fibroblasts)	iCAF: **CXCL12**, S100A6, S100A10, SFRP2,SFRP4,IGF1, CXCL14, ANGPTL4, **IL6**, C3 , CFB , SERPING1	(Shih et al, 2018)
HGSOC	NA	Ascites fluid	iCAF (clusters 8,9), non-iCAF (clusters 6, 7)	iCAF: C1QA/B/C, CFB, CXCL1, CXCL2, CXCL10, **CXCL12, IL6,** IL10	(Izar et al, 2020)
HGSOC (n=1)<br>LGSOC (n=1)	NA	Solid tumor	myCAF (CAF1), mCAF (CAF2)	CAF1: RGS5, HSP90AA1, DNAJA1, PPP1R15A, DNAJB1, FOS, HSPA1B, HSPA1A, MAP1B, HSPB1, HSPD1, HSPA8, HSPE1, HSPA6, ANGPT2, CAV1, PLXDC1, PDGFA, ENPEP, GJC1, NR2F2, JAG1, LAMA4, EPAS1, MEF2C, UBB, PHLDA1, NR4A1, DLC1, ZAK, BTG2, UACA, CRYAB, MYC, BTG1, CDKN1A<br>CAF2: DCN, FN1, COL8A1, LUM, COL1A1, COL11A1, COL1A2, COL3A1, COL6A3, COL10A1, FBLN1, COL5A1, SPON1, COL12A1, COL5A2, COL16A1, COL6A1, FBN1	(Kan et al, 2022)
HGSOC	NA	Ascites fluid	iCAF (iCAF1 & 2), myCAF (myCAF1 & 2)	iCAF1: RPL9^{low}, NME1-NME2^{low}, RPS10^{low}, RPL21^{low}, RPL17^{low}<br>iCAF2: IGLL5, EEF1G, RPS26, RPL21, RPL36A<br>myCAF1: MT-CO3^{low}, MT-ND1^{low}, MT-CO2^{low}, MALAT1^{low}, C1R^{low}, MT-ND4^{low}<br>myCAF2: HBB, CLDN4, IFI44L, LAMC2, HBA2, CCNA1	(Carvalho et al, 2022)
NA	NA	Solid tumor	paCAF, iCAF, myCAF	paCAF: COL1A1, FAP, PDPN, DCNVIM<br>iCAF: **IL6**, PDGFRA, **CXCL12**, CFD, DPT, LMNA, AGTR1, HAS1, CXCL1, CXCL2, CCL2, IL8<br>myCAF: ACTA2, TAGLN, MMP11, MYL9, HOPX, POSTN, TPM1, TPM2	(Yu et al, 2022)
HGSOC	IIIB (n=1), IIIC (n=2)	Solid tumor, ascites fluid	mCAF, iCAF, myCAF, STAR^{+} CAF	mCAF: COL11A1, MFAP5, SFRP2, ISLR, COL10A1, RGCC, MMP11, CTHRC1, IGF2, DERL3<br>iCAF: VTN, SBSN, CADM3, PLIN2, SERPINB2, C3, PTGIS, RP11-572C15.6, KRT19, KRT8, KRT18<br>myCAF: COX4I2, HIGD1B, PTP4A3, RGS5, KCNJ8, TFPI, MCAM, PPP1R14A, NDUFA4L2, TPPP3<br>STAR^{+} CAF: ACTA2^{low}, FAP^{low}, PDPN^{low}, STAR, IGFBP5, TSPAN8, C7, ALDH1A1, LGR5, COLEC11, RNASE1, CADPS, FABP5, HOPX	(Loret *et al* , 2022)
HGSOC	Late stage HGSOC	Ascites fluid	mCAF	mCAF: PTHLH, FGF1, WNT7B, WNT2, TGFB3, MMP11, THRC1, POSTN, VCAN, COL10A1	(Xu *et al* , 2022)
HGSOC	IIIC (n=3), IVA (n=7), IVB (n=1)	Solid tumor	mCAF (CAF-1), iCAF (CAF-2), myCAF (CAF-3)	CAF-1: MMP1,9,10,14<br>CAF-2: LIF, **IL6**, **CXCL12**, CFD<br>CAF-3: ACTA2, MYL9, POSTN, TAGLN	(Zhang *et al* , 2022)
High-grade serous tubo-ovarian cancer	IIIC (n=2), IVB (n=4)	Solid tumor	myCAF (FB_MYH11, FB_RGS5)<br>mCAF (FB_COL27A1, FB_COMP, FB_SERPINE1)<br>iCAF(FB_CALB2, FB_CFD)	FB_MYH11: MYH11, PLN, ADIRF, RERGL, TAGLN, PPP1R14A, MYL9, DSTN, BCAM, SOD3<br>FB_RGS5: NDUFA4L2, HIGD1B, COX4I2, CCDC102B, AVPR1A, TRPC6, GJC1, ADAP2, FAM162B, CD36, RGS5<br>FB_COL27A1: MMP11, POSTN, CTHRC1, COL6A1, COL5A2, COL1A1, MFAP2, LGALS1, MMP23B, ISLR<br>FB_COMP: COL10A1, COL11A1, SERINC2, INHBA, SUGCT, EPYC, CTHRC1, FN1, COMP, RARRES2<br>FB_SERPINE1: CADM3, APOE, SERPINE1, PTGIS, EGFL6, ANGPTL4, LXN, SAT1, CLDN1, RARRES1<br>FB_CALB2: ITLN1, HP, PLA2G2A, PRG4, MSLN, TFPI2, UPK3B, CALB2, SLC39A8, CLDN1<br>FB_CFD: PI16, C16orf89, SCARA5, ITM2A, F10, TNXB, CFD, CD34, WISP2, FBLN5	(Obrecht *et al* , 2020)
HGSOC	IIC (n=1), IIIC (n=1)	Solid tumor	iCAF-like STAR^{+} CAF (Fibro_1)<br>mCAF (Fibro_2)<br>apCAF (Fibro_3, Fibro_5)<br>TNF-related / iCAF-like (Fibro_4)	Fibro_1: STAR, ZNF331, ADAMTS4, CEBPD, CYCS, MEG3, EIF4A3, ATP1B3, AREG, IQCG<br>Fibro_2: CTHRCI, MMP11, COL1A1, COL3A1, VCAN, COL1A2, FN1, SPARC, HOPX, IGFBP2<br>Fibro_3: OGN, CST3, CFD, PLA2G2A, C3, MGP, GSN, CCDC80, APOD, IGFBP6<br>Fibro_4: FOS, JUN, EGR1, IER2, DNAJB1, HSPA1B, HSPA1A, GADD45B, KLF2, SOCS3<br>Fibro_5: MALAT1, XIST, NEAT1, KDM6B, ABL2, KCNQ1OT1, CFLAR, XAF1, PARP14, SOD2	(Deng *et al* , 2022)
**PSM-Gastric cancer**					
Gastric adenocarcinoma	Intestinal (based on Lauren's classif.)	Solid tumor	iCAF (F0), myCAF (F1)	F0: **CXCL12**, IL7R, RGS1, CXCR4, CD55, COL6A3, IL32, DCN, SRGN, ARL4C, COL1A1, S100A10, FBLN5, CFD, COL1A2, OGN, FBN1, CTSK, PLXDC2, C3<br>F1: BCAM, MCAM, TINAGL1, TAGLN, IGFBP7, C11orf96, ADIRF, MYL9, SPARCL1, NOTCH3, MYH11, CAV1, MYLK, CSRP2, EPAS1, DSTN, TPM2, ACTA2, NDUFA4L2, RGS5	(Jiang et al, 2022)
Gastric adenocarcinoma	Diffuse (n=20) Intestinal (n=2) (based on Lauren's classif.)	Ascites fluid	iCAF & iCAF-like(fibro_c1, fibro_c15, fibro_c6, fibro_c9)<br>mCAF (fibro_c2)	fibro_c1: CFD, FBLN1, CCDC80, C7, GSN, MFAP5, PI16, DCN, IGFBP6, MGP, SFRP2, OGN, PLAC9, ADH1B, SFRP1, C1QTNF3, SERPINF1, OMD, CST3, PRELP, IGF1, GPNMB, C1R, TNXB, CLU, FBLN2, SFRP4, APOD, UAP1, MT1X<br>fibro_c15: IL11, CXCL8, CXCL1, MMP1, **CXCL3**, COL7A1, CHI3L1, TNFAIP6, TNC, SOD2, TFPI2, FTH1, G0S2, TMEM158, INHBA, CD44, SDC4, CXCL2, CCL2, PTGS2, **IL6,** SERPINE1, PLAU, THBS1, IER3, GEM, MT2A, CTSL, LMCD1, NFKBIA<br>fibro_c6: CXCL14, VSTM2A, NSG1, APOD, ENHO, POSTN, LSP1, BMP4, PDGFRA, MFAP4, CPM, TRPA1, HSD17B2, DPT, F3, TMEM176B, IGFBP5, RBP4, AGT, PLAT, EDNRB, MMP2, HAAO, PDGFD, SOX6, RGS10, CXCR4, TMEM176A, TSPAN12, GADD45G<br>fibr_c9: NPY, PTGS1, NRG1, AREG, HSD17B2, FENDRR, OTULINL, CXCL14, APOC1, AGT, NSG1, ACP5, APOE, RGS10, F3, TMEM176B, DIO2, TMEM176A, GDF15, PLAU, PDGFRA, MFGE8, PLAT, POSTN, ADM, TCIM, IGFBP3, PTN, TGFBI, DMKN<br>fibro_c2: COL1A1, COL1A2, COL3A1, CTHRC1, ASPN, GREM1, COL5A2, INHBA, LUM, COL12A1, COL6A3, THBS2, COL10A1, VCAN, COL11A1, CTSK, MMP11, SPON2, ANTXR1, RARRES2, AEBP1, TGFBI, PRRX1, BGN, SULF1, FN1, RAB31, SPARC, APOE, CCL11	(Wang et al, 2023)

Suppl. Table 1. Fibroblast subtypes in peritoneal surface malignancies. Wherever applicable, originally reported fibroblast subtype group names are shown in parentheses.

Abbreviations: HGSOC: High-grade serous ovarian cancer, LGSOC: Low-grade serous ovarian cancer, myCAF: myofibroblastic CAF, iCAF: inflammatory/immune-regulatory CAF, mCAF: matrix CAF, paCAF: perpetually activated CAF, apCAF: antigen presenting CAF
